# Design approach of an aquaculture cage system for deployment in the constructed channel flow environments of a power plant

**DOI:** 10.1371/journal.pone.0198826

**Published:** 2018-06-13

**Authors:** Taeho Kim, Jihoon Lee, David W. Fredriksson, Judson DeCew, Andrew Drach, Solomon C. Yim

**Affiliations:** 1 Division of Marine Technology, Chonnam National University, Yeosu, Republic of Korea; 2 Department of Naval Architecture and Ocean Engineering, United States Naval Academy, Annapolis, MD, United States of America; 3 Center for Ocean Engineering, University of New Hampshire, Durham, NH, United States of America; 4 Institute for Computational Engineering and Sciences, University of Texas at Austin, Austin, TX, United States of America; 5 School of Civil and Construction Engineering, Oregon State University, Corvallis, OR, United States of America; Coastal Carolina University, UNITED STATES

## Abstract

This study provides an engineering approach for designing an aquaculture cage system for use in constructed channel flow environments. As sustainable aquaculture has grown globally, many novel techniques have been introduced such as those implemented in the global Atlantic salmon industry. The advent of several highly sophisticated analysis software systems enables the development of such novel engineering techniques. These software systems commonly include three-dimensional (3D) drafting, computational fluid dynamics, and finite element analysis. In this study, a combination of these analysis tools is applied to evaluate a conceptual aquaculture system for potential deployment in a power plant effluent channel. The channel is supposedly clean; however, it includes elevated water temperatures and strong currents. The first portion of the analysis includes the design of a fish cage system with specific net solidities using 3D drafting techniques. Computational fluid dynamics is then applied to evaluate the flow reduction through the system from the previously generated solid models. Implementing the same solid models, a finite element analysis is performed on the critical components to assess the material stresses produced by the drag force loads that are calculated from the fluid velocities.

## Introduction

Because of the expansion of aquaculture along the coastline, it is essential to consider systems that serve multiple purposes and to develop innovative approaches that minimize spatial conflicts. Specifically, utilizing the existing infrastructure to maximize fish production is an important step towards achieving efficient processes from both a biological and economic perspective. The aquaculture potential offered by the thermal discharges of power stations is widely recognized [1]. Their suitability for fish farming largely depends on the cooling water sources and on the design and operation of the power station [2]-[4]. Water of varying quality and salinity is used to generate steam and drive the turbines, which then drive the generators, whereas the waste steam is fed to a condenser and discharged as thermal effluent [1]. The heated water that is discharged from the steam-electric generating stations has been considered to be a nuisance and a direct threat to aquatic life [2].

Although efficient water exchange is essential for the replenishment of oxygen and the removal of waste metabolites, the water current influences the behavior of fish, which affect the social hierarchies, growth, and growth disparities among the stock. Excessive currents impose additional dynamic loads to the cage, support structures, and moorings, which may adversely affect the behavior of fish and contribute to the food losses of semi-intensive and intensive operations. The water current velocities in thermal effluent canals can be highly variable. Generally, it is recommended that the current velocities at the cage sites remain less than 0.6 m/s [1], as high currents can load to the deformation of net cages, excessive strain on mooring and cage collars, and unacceptable losses of feed and waste.

**Fig 1:**
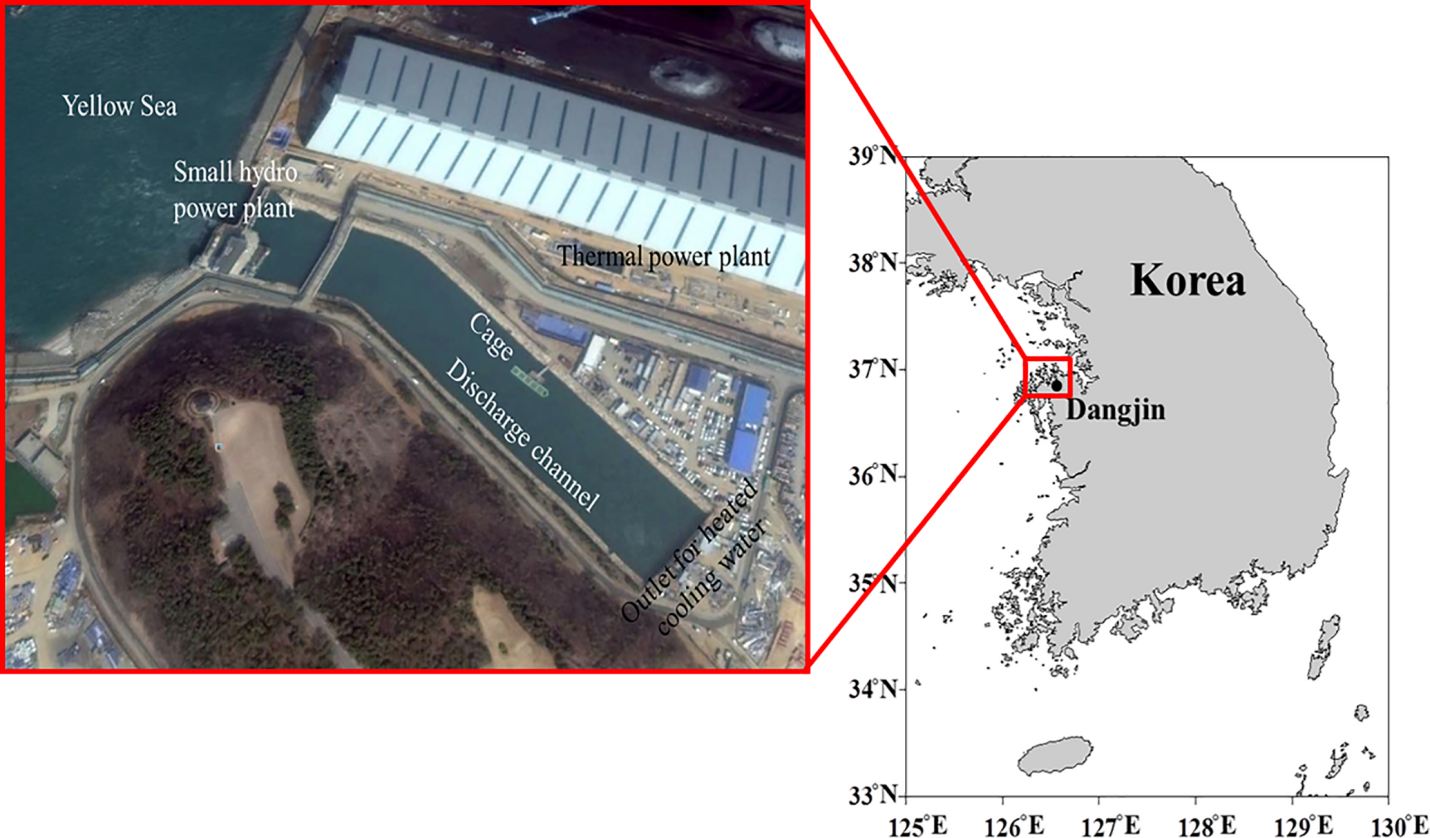
Location of the coolant water discharge channel of a coal-fired power plant in Dangjin, South Korea.

Fig 1 shows the location of the coolant water discharge channel of a coal-fired power plant (containing 6000 MW turbines) in Dangjin, South Korea. As shown in Fig 1, a small hydroelectric power plant is installed at the end of the discharge channel to maintain a constant water level. The water temperature in the surrounding region is dependent upon the location, water supply, design of the system, site (canal), and proximity of the cages to the power station. The coolant water from the power plant that is used to condense the steam (which is contained within an isolated circuit) is released into the constructed channel. Because the warm temperature of the discharged water is predictable, it may be feasible to choose an appropriate aquaculture product to maximize growth rates in this environment. Maximizing the growth rates, in turn, maximizes the potential for economic viability. However, strong currents within the discharge channel can impose large loads on the containment structures and cause excessive stress to fish because of the increased energy used for swimming to maintain position.

In this study, an aquaculture system for deployment in a power plant discharge channel was designed using computer-aided design (CAD) software. In order to maintain an adequate supply of protein for the growing world population, the importance of seafood production through sustainable aquaculture practices is becoming more evident. Additionally, as the aquaculture industry expands, it is increasingly important to develop novel engineering design techniques. Recently, the use of “off-the-shelf” computer modeling tools is becoming more prevalent in the design and analysis of marine aquaculture structures. One of the first applications was presented by Gignoux et al. [5], where the concept of a mapping coefficient with beam elements was introduced using a version of ABAQUS to represent the dense net meshes with fewer elements. Helsley and Kim [6] performed computational fluid dynamics (CFD) simulations using the FLOW3D finite difference code for an implemented downstream diffusion of a bi-conical rigid cage system. The results indicated that, even at small angles of tilt, enhanced mixing occurred. Fredriksson et al. [7] applied structural analysis techniques using the MSC Marc finite element modeling (FEM) software to evaluate the use of high-density polyethylene plastic pipes in marine applications. To investigate the potential design procedures for marine-deployed closed containment aquaculture systems, Fredriksson et al. [8] applied CFD using the Fluent 14.0 (ANSYS, Inc.) software to simulate the hydrodynamics surrounding closely spaced solid cylinders. This study also utilized the MSC Marc software to investigate the structural characteristics of a potential system design. In Zhao et al. [9], a model of a three-dimensional (3D) net was established using the lumped mass method. Patursson et al. [10] also applied CFD using the Fluent software to analyze the flow-through of net panels with a porous media model. A screen-type force model for the viscous hydrodynamic load on the nets was proposed by Kristiansen and Faltinsen [11]. Oh et al. [12] performed numerical modeling techniques using two separate FEM software packages to analyze a submersible sea cucumber cage. In Zhao et al. [13], a 3D numerical model was established to simulate the flow field inside and around the gravity cages that were exposed to a current. Kim et al. [14] conducted CFD and FEM modeling to analyze the flow characteristics through a submersible abalone cage system and to assess its structural characteristics. Kim et al. [15] also investigated the dissolved oxygen and animal survival and growth in co-culture cage systems for the grow-out of juvenile abalone (*Haliotis discus hannai*) with juvenile sea cucumber (*Apostichopus japonicas*, Selenka) using CFD analysis and indoor seawater tanks.

In this paper, the design of a commercial-size canal cage system with a nose cone structure is analyzed for implementation in the constructed channel flow environment of a power plant. Since strong currents can impose large loads on the containment structures and cause excessive stress to fish, flow availability is a substantial design issue. Thus, numerical modeling analyses were performed using CFD software to determine the adequate flow characteristics through the cage system. The structural model program, MSC Marc, was configured to represent the cage structure for analyzing the fluid forces and performing the stress calculations. The FEM technique was used again, but with SolidWorks, to perform detailed calculations at the critical locations determined by the global stress data set.

## Channel characterization

**Fig 2:**
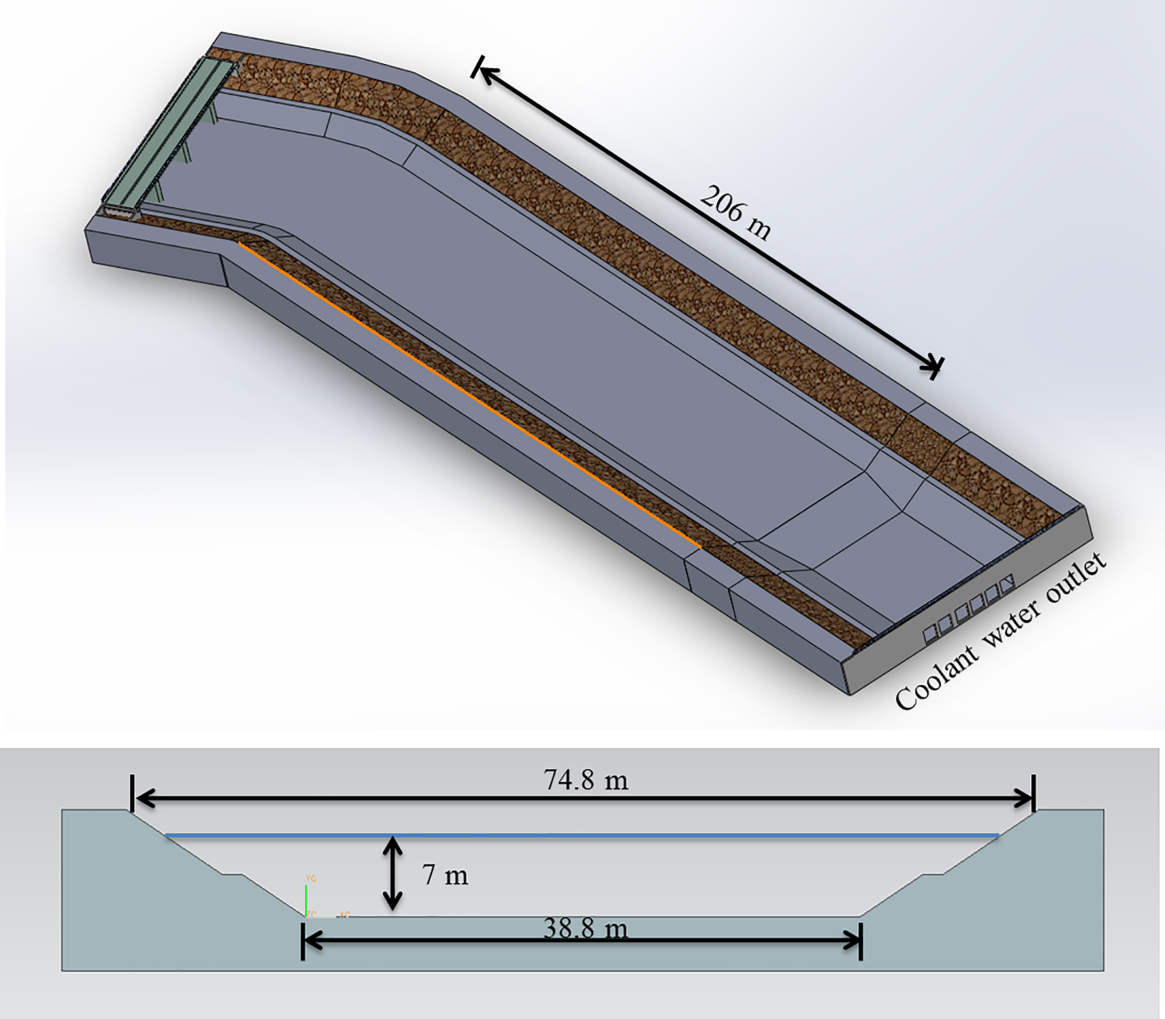
Dimensions of the coolant water discharge channel.

Fig 2 shows a 3D image of the coolant water discharge channel. The length where the cage system is deployed in the discharge channel is approximately 206 m. In addition, Fig 2 shows that the channel has a trapezoidal shape with surface and bottom widths of 74.8 m and 38.8 m, respectively, and a channel depth of 7.0 m. Measurements of the various channel conditions were taken periodically to gain a understanding of the seasonal impact. The flow velocity of the channel was measured as 0.20 m/s in May and 0.57 m/s in August 2014. The maximum water temperature of the water was 28.76°C at the end of August, and the lowest water temperature was 12.44°C in the winter of February 2014. A water temperature of 28°C, which is the water temperature limit for most species of fishes, was present from July to September over a period of three months. From January to December 2014, the salinity ranged from 30.44 psu to 32.12 psu, the dissolved oxygen ranged from 6.85 ppm to 10.86 ppm, and the pH ranged from 7.62 to 8.0, respectively. The maximum thermal effluent temperature is 10°C warmer than that of the intake (i.e., thermal power stations have a Δ*T* of 10°C). The survival rates of caged fish in discharge canals have been extremely poor [3], [4], [16], and thus, it is necessary to increase the water temperature gradually for minimum of 30 days in an adjustable temperature aquarium prior to stocking the fish in the general marine cage or aquaculture tank.

## System description

**Fig 3:**
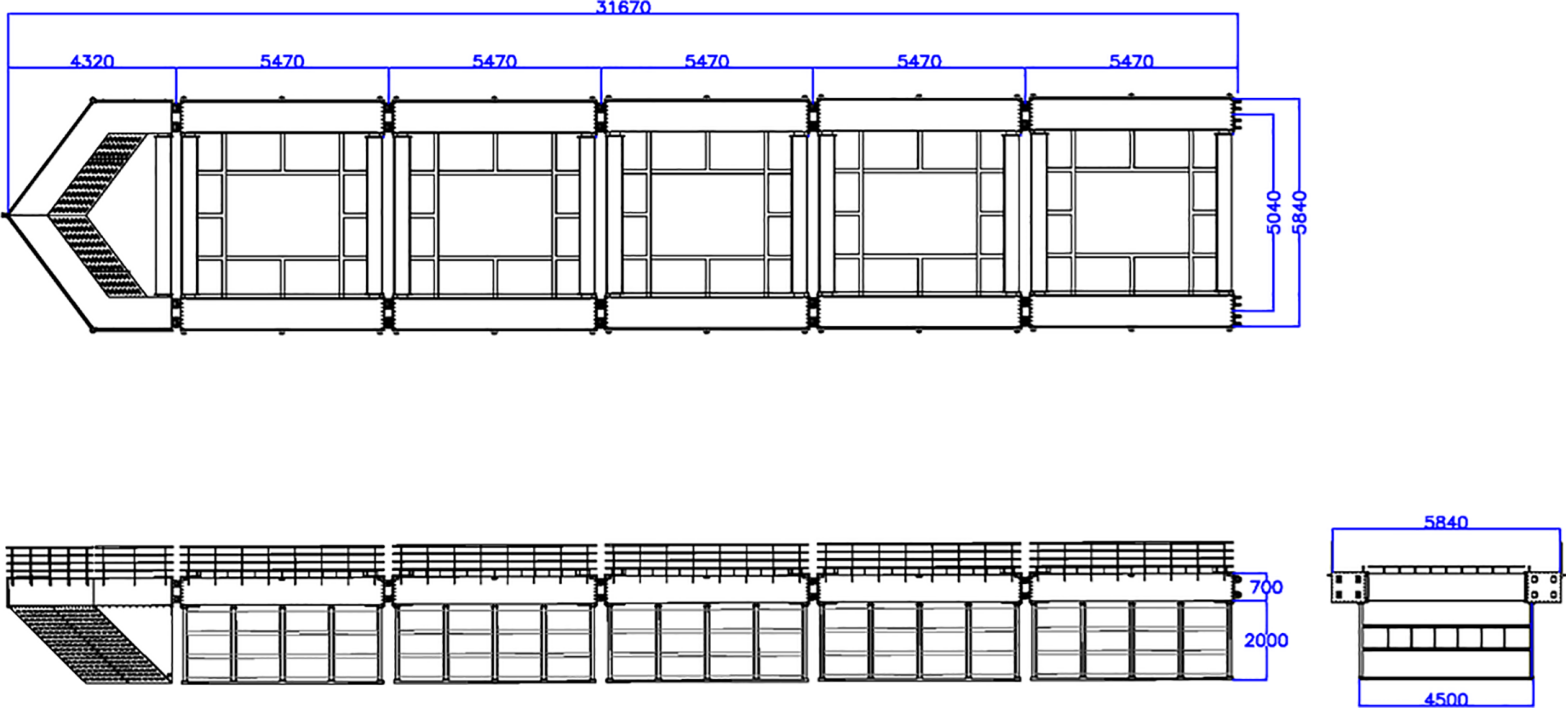
Rigid cage system consisting of five modular components, a perforated nose cone, and side flow velocity reduction devices (unit: mm).

As previously described, an engineering analysis was conducted on segments of the semi-rigid aquaculture system that is designed for deployment within a constructed channel flow environment. Fig 3 shows that the structure consists of a modular assembly encompassing five containment components. Each component has the general dimensions of 5.47 m for the length, 5.04 m for the width, and 2.70 m for the height. The structural materials were assumed to be made of basic carbon steel galvanized with zinc. As shown in Fig 3, the modular assembly includes a nose cone component located on the left side, which is important design feature. The entire system has a length of 31.67 m, including the nose cone portion. The purpose of the nose cone is to reduce the flow velocity by deflecting the flow around the fish containment modules (i.e., fish cage). By deflecting these strong flows, the nose cone effectively reduces the normal velocity components for the downstream fish containment modules. Additionally, the nose cone is perforated due to the importance of water exchange in aquaculture systems. The perforations allow oxygenated water to pass through the system, which aids in maintaining the exchange of suitable dissolved oxygen for the fish to cultivate. Moreover, the side flow velocity reduction devices are also located on both sides of the five containment components to further deflect the flow around the fish containment modules.

## CFD analysis approach

### Flow model

Governing equations. The 3D flow pattern inside and around the constructed channel and the cage system was analyzed with the CFD program, Fluent 14.0, as described in the program manual [17]. Steady-state conditions were assumed for the flow calculations, and the standard *k*–*ε* model was applied as the turbulence model. The continuity and the Reynolds-averaged Navier-Stokes (RANS) formulation were used for the analysis. The turbulent eddy viscosity (*μ*_*t*_) was determined by applying turbulence in the standard *k*–*ε* model, as shown in Eq (1).

$$\mu_{t}=\rho C_{\mu }\frac{k^{2}}{\varepsilon }$$ (1)

Where *C*_μ_ is a model constant that is equal to 0.09. In Eq (1), the turbulent kinetic energy (*k*) and the turbulent kinetic energy dissipation rate (*ε*) were simulated by two transport equations, as shown in Eqs (2) and (3), respectively.

$$\frac{\partial }{\partial t}(\rho k)+\frac{\partial }{\partial x_{i}}(\rho ku_{i})=\frac{\partial }{\partial x_{j}}\left[(\mu +\frac{\mu_{t}}{\sigma_{k}})\frac{\partial k}{\partial x_{j}}\right]+P_{k}+P_{b}-\rho \varepsilon -Y_{M}+S_{k}$$ (2)

$$\frac{\partial }{\partial t}(\rho \varepsilon )+\frac{\partial }{\partial x_{i}}(\rho \varepsilon u_{i})=\frac{\partial }{\partial x_{j}}\left[\frac{(\mu +\frac{\mu_{t}}{\sigma_{\varepsilon }})\partial \varepsilon }{\partial x_{j}}\right]+C_{1\varepsilon }\frac{\varepsilon }{k}(P_{k}+C_{3\varepsilon }P_{b})-C_{2\varepsilon }\rho \frac{\varepsilon^{2}}{k}+S_{\varepsilon },$$ (3)

Where, *t* is time, *P*_*k*_ is the turbulent kinetic energy generated by the average velocity gradient; *P*_*b*_ is the turbulence kinetic energy generated due to buoyancy; *Y*_*M*_ is the fluctuating dilatation of compressible turbulence that contributes to the overall dissipation rate, such that *P*_*b*_ = 0 and *Y*_*M*_ = 0; *S*_*k*_ and *S*_*ε*_ are the source terms that can be defined by the user; σ_*k*_ and σ_ε_ are the turbulent Prandtl numbers. The model constants for the realizable *k*–*ε* turbulence model are σ_ε_ = 1.2, *C*_1*ε*_ = 1.44,*C*_2*ε*_ = 1.92,*σ*_*k*_ = 1.0, and $C_{3\varepsilon }=\tanh\left(\frac{u_{p}}{u_{v}}\right)$, where *u*_*p*_ and *u*_*v*_ are the velocity components parallel and normal to gravity, respectively.

Porous model. The 3D flow pattern around the nose cone was analyzed using the CFD program. Fig 3 shows that the nose cone of the channel cage system has a thin perforated plate, on which small holes are densely arranged. However, the shapes should not be simulated using dense grids due to the calculation load, as the amount of flow passing through the holes is extremely small compared with the flow through the channel and the cage system. Therefore, CFD model of the nose cone was conducted using an approximation method with a porous material on which small holes are evenly arranged. This routine applied the finite volume method to solve the governing equations.

The permeable walls of the nose cone (consisting of a mesh-like structure) were represented using the porous media model described in Patursson et al. [10]. The flow through the nose cone is described using the equation for flow through a porous media, as shown in Eq (4).

$$f_{i}=-(D_{ij}\mu u_{j}+C_{ij}\frac{1}{2}\rho |u|u_{j}),$$ (4)

Where *D*_*ij*_*μu*_*j*_ and $C_{ij}\frac{1}{2}\rho |u|u_{j}$ are the viscous loss term and the inertial loss term, respectively. *D*_*ij*_ and *C*_*ij*_ are the prescribed matrices consisting of the porous media resistance coefficients presented in Eq (5).

$$D_{ij}=\left[\begin{array}{ccc} D_{n} & 0 & 0\\ 0 & D_{t} & 0\\ 0 & 0 & D_{t} \end{array}\right],\;C_{ij}=\left[\begin{array}{ccc} C_{n} & 0 & 0\\ 0 & C_{t} & 0\\ 0 & 0 & C_{t} \end{array}\right]$$ (5)

Where, *D*_*n*_ is a normal viscous resistance coefficient; *D*_*t*_ is the tangential viscous coefficient; *C*_*n*_ is the normal inertial resistance coefficient; *C*_*t*_ is the tangential inertial resistance coefficient.

The normal inertial resistance coefficient can be calculated for the pressure loss through a perforated plate with holes using an empirical equation that derives the porous media inputs for turbulent flow through a perforated plate or net. The relationship between the mass flow rate ($\dot{m}$) through the porous material and pressure drop [18] is described in Eq (6).

$$\dot{m}=CA_{f}\sqrt{\left(2\rho \Delta p/[1-(\frac{A_{f}}{A_{p}})\right]}.$$ (6)

Where *A*_*f*_ is the free area or the total area of the holes; *A*_*p*_ is the area of the nose cone and netting (including the solid area and the area of the holes); *C* is an experimental coefficient equal to 0.98, as described in Smith and Winkle [19].

Because $\dot{m}=\rho uA_{p}$, Eq (6) can be rewritten, as shown in Eq (7).

$$\Delta p=\left(\frac{1}{2}\rho u^{2}\right)\frac{1}{C^{2}}[{\left(\frac{A_{p}}{A_{f}}\right)}^{2}-1].$$ (7)

In the case of netting, the pressure drop is mainly attributed to the inertial loss term, and thus, *C*_2_ is represented in Eq (8).

$$C_{2}=\frac{1}{C^{2}}[{\left(\frac{A_{p}}{A_{f}}\right)}^{2}-1]/T$$ (8)

Where *T* is the thickness of the material.

## CFD analysis

### CFD analysis of the constructed channel

**Fig 4:**
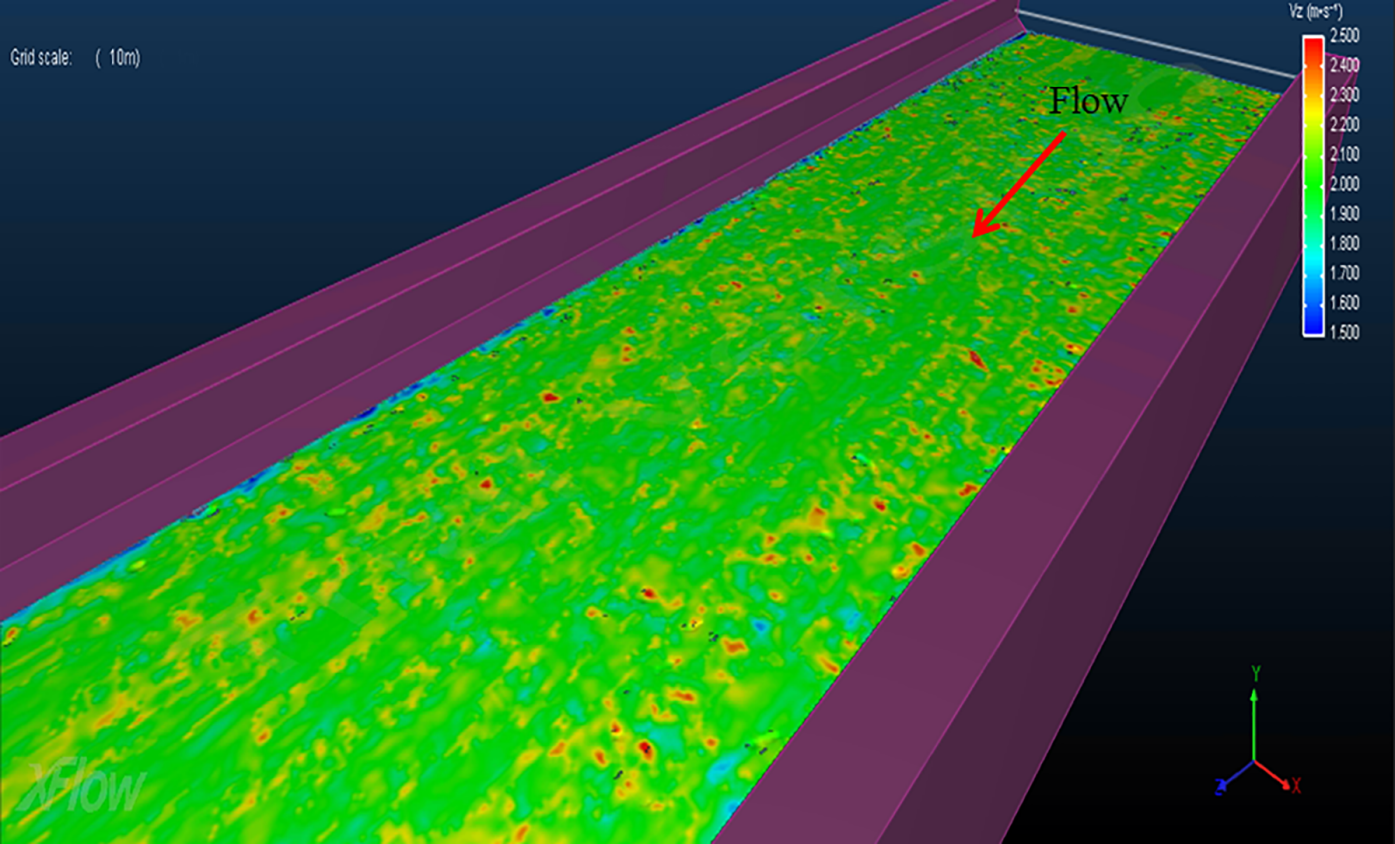
Velocities computed for the cross-section located at a distance of 206 m downstream of coolant water outlet.

Prior to the design of the cage system, the monthly flow rates of the discharge channel were calculated to analyze the design flow velocities and flow characteristics of the constructed channel. Table 1 present the average monthly flow rates and the corresponding inlet flow velocities from 2010 to 2011. Using the data from Table 1, the average inlet flow rate was 0.66 m/s in August and 0.27 m/s in May. The inlet flow rates were calculated for use in the flow velocity and flow distribution calculations. Thus, assuming that the displacement is constant on a monthly basis, and the water level remains constant, the inlet flow velocity can be calculated by dividing the displacement by the cross-sectional area of the channel. Fig 4 shows the velocities computed for the cross-sectional area located 206 m downstream from the coolant water outlet. Based on the inlet flow velocity, the flow velocity distribution and the maximum flow velocity of the channel can be determined and a simulation was conducted for four cases: the smallest flow rate (inlet flow velocity of 0.27 m/s) in May, the largest flow rate (inlet flow velocity of 0.66 m/s) in August, and inlet flow velocities of 1.0 m/s and 2.0 m/s.

**Table 1:** Flow velocities based on the monthly cooling water flow rate and CFD analysis of the discharge channel.

Month	Flow rate (m^3^/month)	Average inlet flow velocity (m/s)	Maximum flow velocity from CFD analysis (m/s)
January	206,965,882	0.32	0.42
February	180,149,361	0.31	0.41
March	195,804,377	0.30	0.41
April	171,838,195	0.28	0.38
May	176,298,650	0.27	0.38
June	211,127,884	0.34	0.44
July	307,002,257	0.48	0.59
August	414,185,396	0.66	0.80
September	396,897,949	0.64	0.76
October	345,851,598	0.54	0.66
November	225,837,917	0.36	0.47
December	207,433,711	0.32	0.43

**Fig 5:**
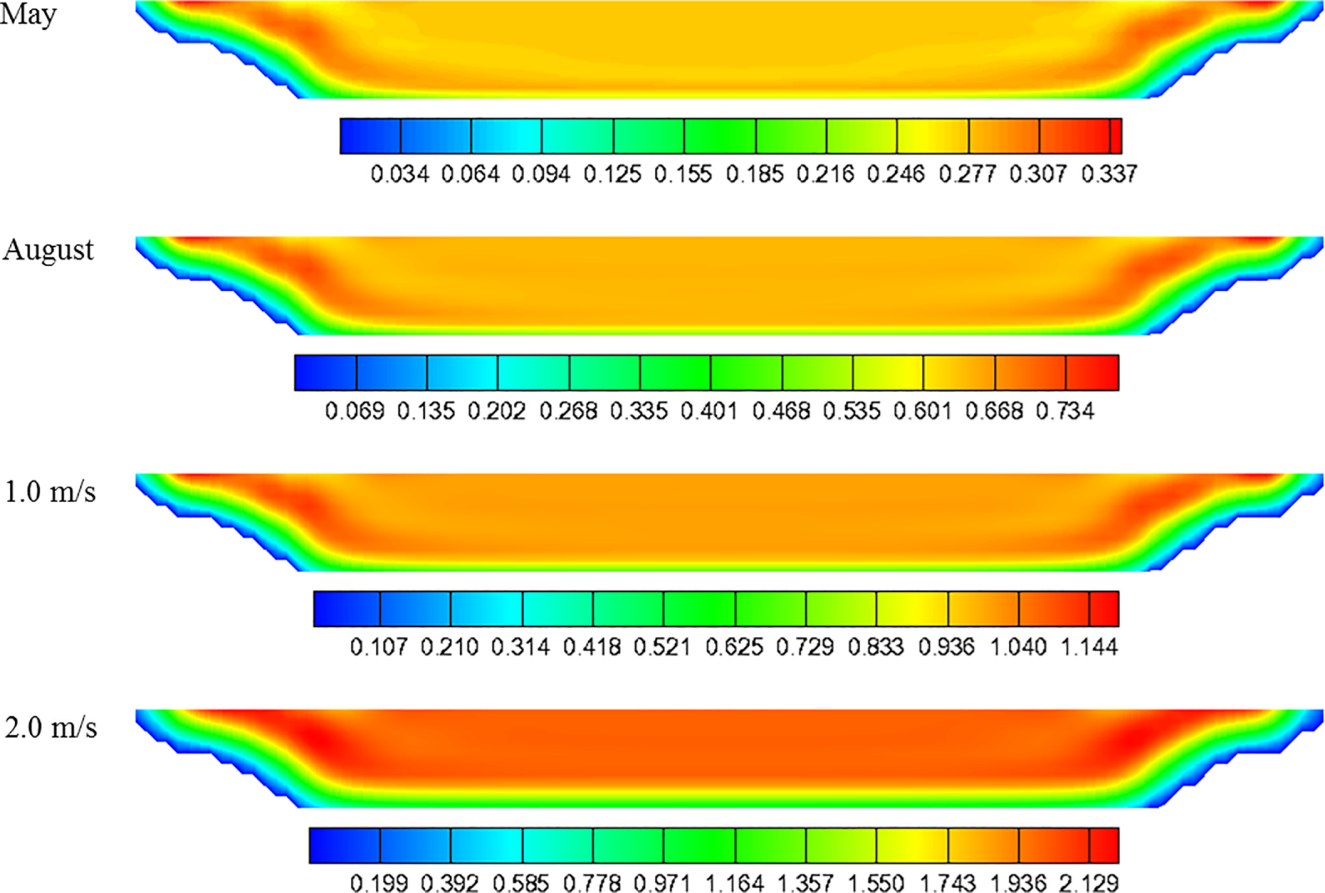
Average flow velocity contour of the coolant water channel.

Fig 5 shows the flow rate distributions of the channel for each case. In Fig 5, the red color indicates fast flow velocities, whereas the blue color indicates slow flow velocities. Fig 5 demonstrates that, as the inlet flow velocity increases, the flow rate in the discharge channel correspondingly increases; however, the distribution pattern of the flow rate does not change significantly.

**Fig 6:**
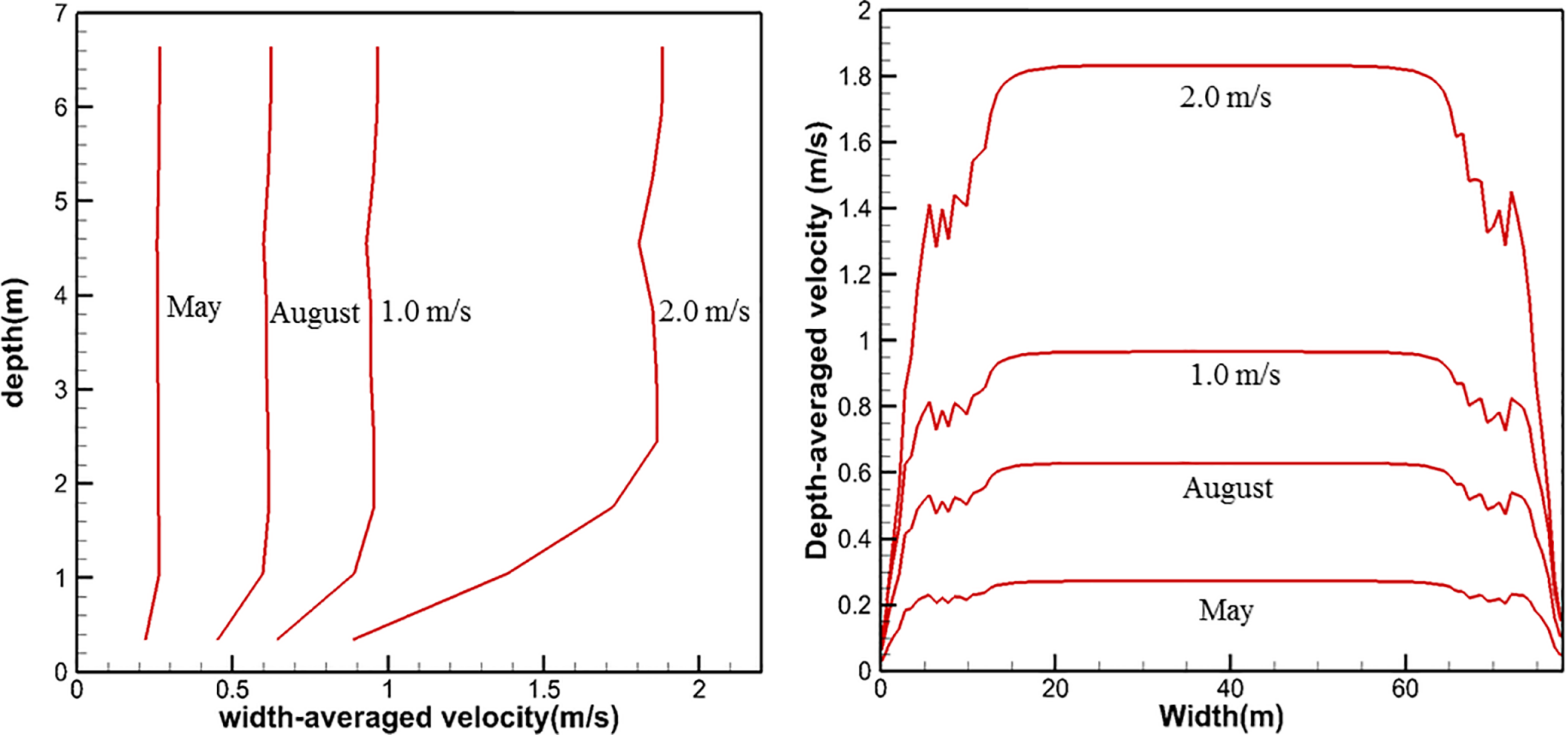
Flow velocity profiles of the channel in the directions of water depth and width.

Fig 6(A) shows the variation of the flow velocity in relation to with the depth of the channel. The flow velocity was averaged in the direction of the channel width from the same height, assuming that the bottom has a height of 0.0 m. As shown in Fig 6(A), all of the cases indicate that the flow velocity remains relatively constant at distances greater than 2.0 m from the floor, regardless of the water depth. Fig 6(B) shows the velocity distribution along the direction of the channel width. In this case, the flow velocity was averaged over the depth at the same location. As shown in Fig 6(B), the flow velocity remains constant between 20 m and 60 m of width for all calculation conditions.

**Fig 7:**
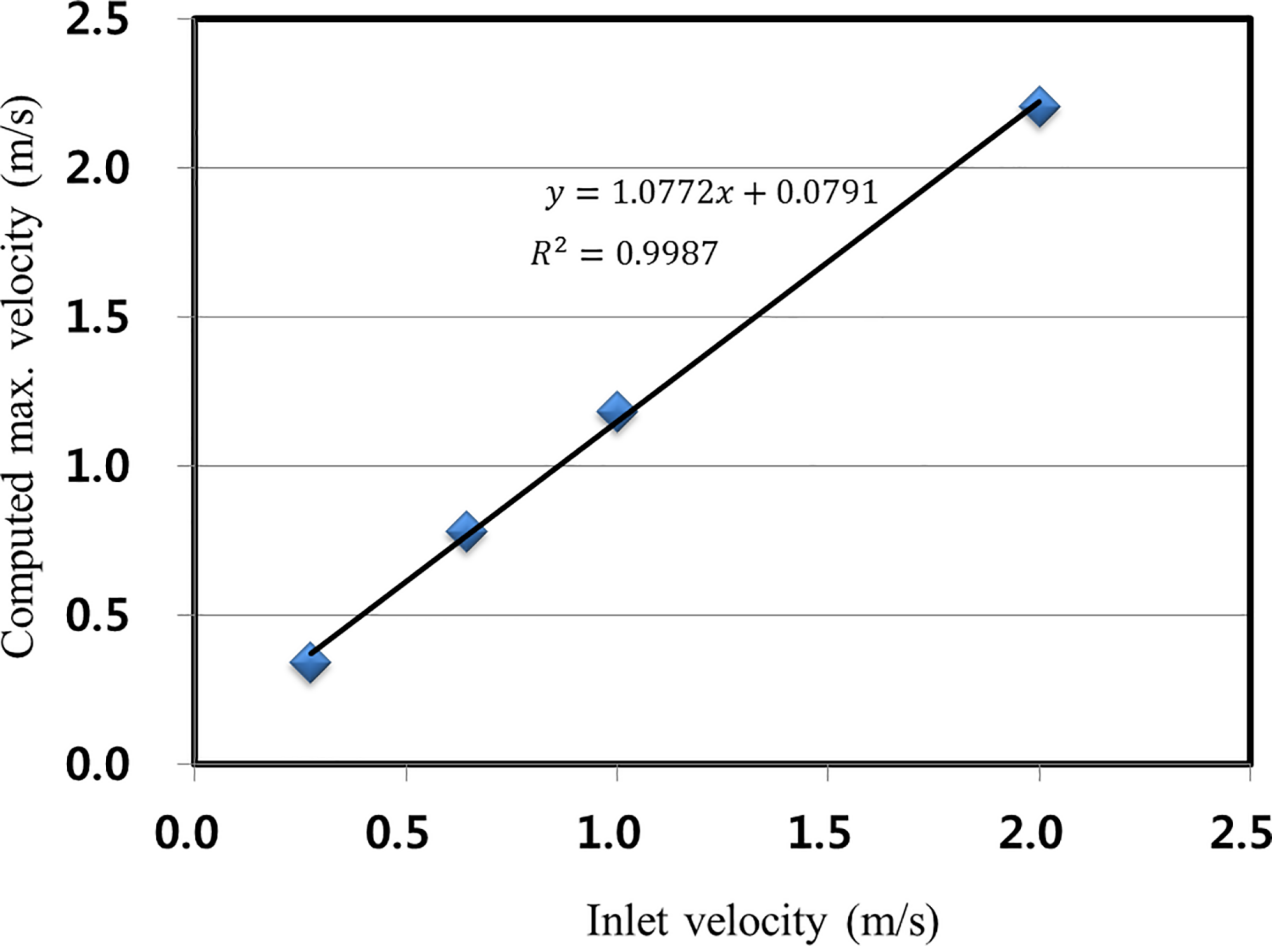
Relationship between the inlet flow velocity and simulated maximum flow velocity obtained by the least-squares method.

The last column of Table 1 presents the simulated maximum flow velocity. As the inlet flow velocity increases, the maximum flow velocity correspondingly increases with a ratio of approximately 1.1–1.25. This ratio indicates that the maximum flow velocity in the channel increases by 10% to 25% of the inlet flow velocity. Fig 7 shows that a linear relationship between the inlet flow velocity (*X*) and the simulated maximum flow velocity (*Y*) can be obtained using a least-square linear fit. The slope of the linear relationship is 1.0722, the intercept was 0.0791, and the correlation coefficient (*R*^2^) is 0.9987, which indicates a near-perfect fit. According to Table 1, the month of May has the lowest maximum flow velocity with a value of 0.38 m/s (corresponding to an inlet flow velocity of 0.27 m/s). In contrast, the month of August has maximum flow velocity with a value of 0.80 m/s (corresponding to an inlet flow velocity of 0.66 m/s).

In order to determine the maximum flow velocity capable of being generated in the discharge channel, it is assumed that the discharge flow rate will increase with the enhancement of the output due to the completion of the 9^th^ and 10^th^ power generation facilities, which are scheduled to be under construction in 2018. The flow rate was calculated by assuming a 70% flow rate increase. The highest flow velocity for the month of May was calculated as 0.63 m/s (corresponding to an inlet flow velocity of 0.47 m/s), which is the lowest flow rate. The highest flow velocity was obtained for the month of August as 1.31 m/s (corresponding to an inlet flow velocity of 1.09 m/s). Therefore, in this study, the maximum flow rate of 1.31 m/s was applied as the flow rate of 170% for the flow analysis of the cage system, including that without any structure within the channel, the “free-stream” flow was assumed to be 1.31 m/s.

### CFD analysis of a perforated nose cone

**Fig 8:**
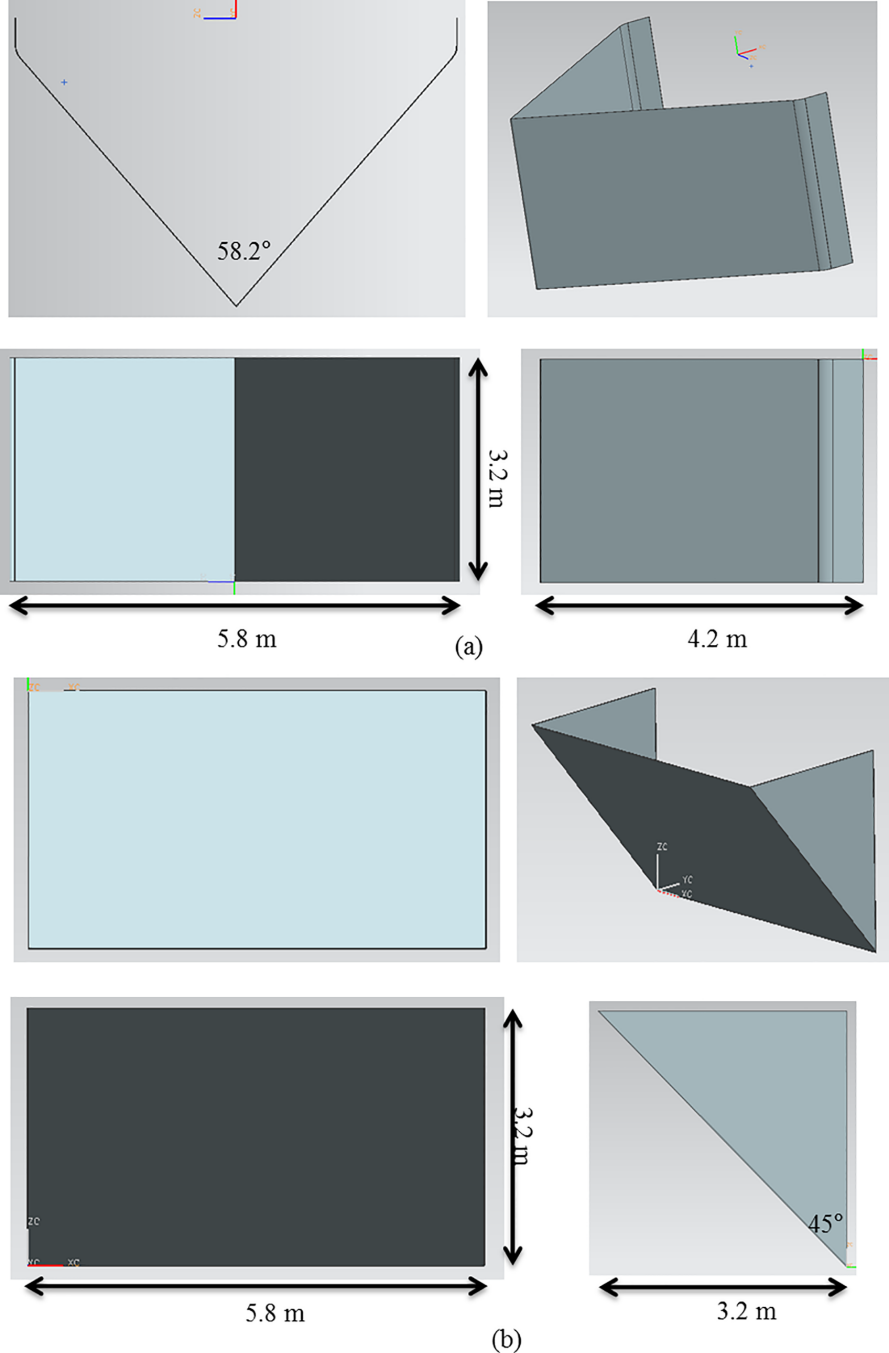
Nose cone shapes used in CFD analysis: (a) Case 1 and (b) Case 2.

In order to determine the proper shape and porosity of the front flow reduction device for the discharge channel cage, the flow characteristics of two types of nose cones were analyzed using CFD; Case 1 and Case 2. Based on the CFD results, the optimum slope angles of the nose cones for Case 1 and Case 2 were determined. Fig 8(A) shows the nose cone shape of the channel cage for Case 1. The design of the Case 1 nose cone had a slope of 45°, height of 3.2 m, and width of 5.8 m. This design allowed the structure to maintain buoyancy of the nose cone. Fig 8(B) shows the nose cone shape of the channel cage for Case 2. In this case, the perforated plate was a V-shape, and the angle of the two front faces was designed to be 58.2° with height of 3.2 m and a width of 5.8 m.

**Fig 9:**
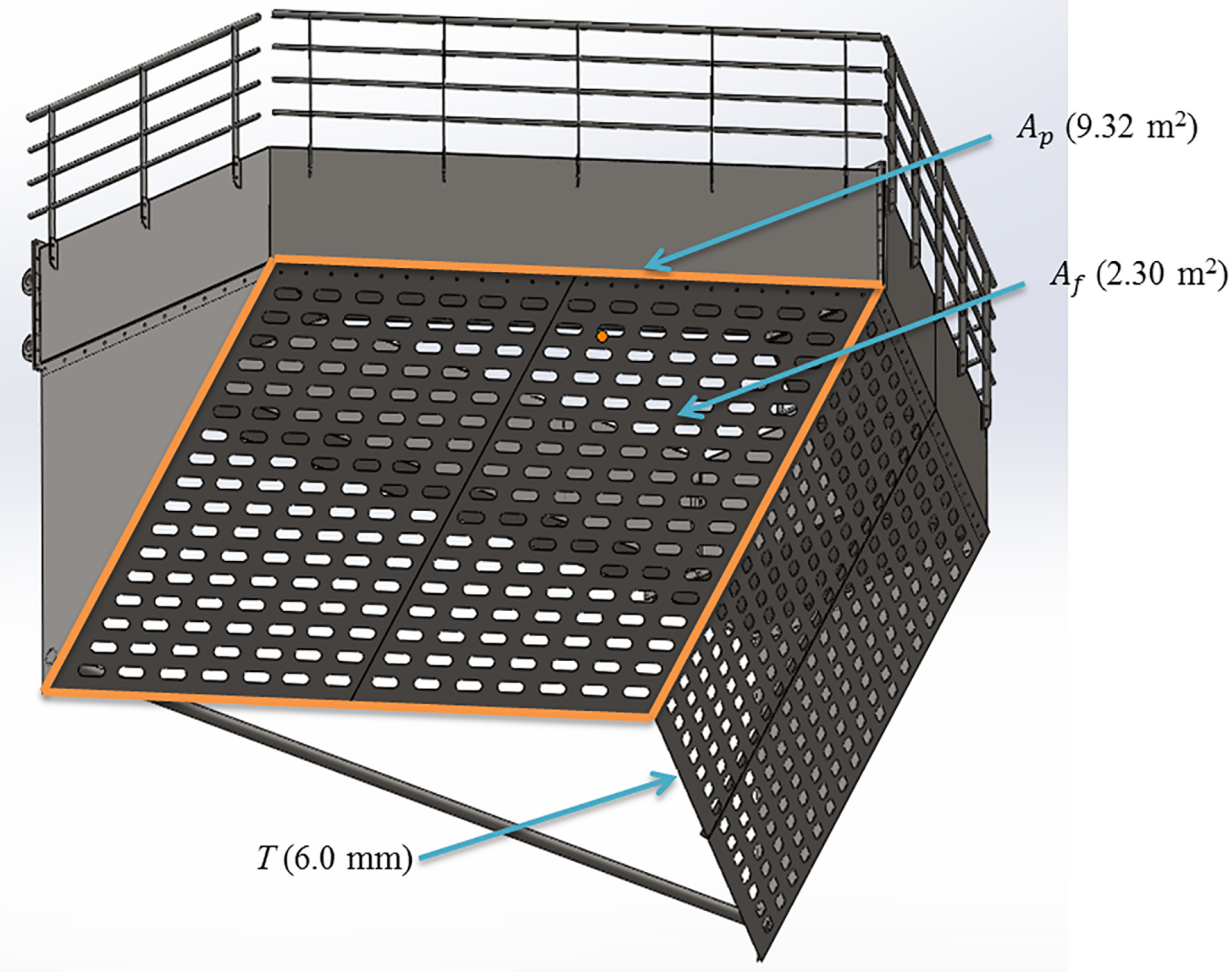
Free area (*A*_*f*_) and total area (*A*_*p*_) of a perforated nose cone.

Fig 9 shows the actual shape of the nose cone. The nose cone was modeled as a porous material. The porosities of the nose cone were determined by the rates between the total area of the holes (*A*_*p*_) and the free area of the holes (*A*_*f*_), indicating that the number of holes in the nose cone was not defined. In order to evaluate the flow characteristics of the nose cone according to the changes in porosity, three porosities (*A*_*p*_/*A*_*f*_) were studied herein: 50%, 33%, and 10%. The example shown in Fig 9 indicates that this particular CFD analysis used an *A*_*p*_ of 9.32 m^2^ and an *A*_*f*_ of 2.30 m^2^. Meanwhile, the length and width of the channel discharge water were defined as approximately 206 m and 74.8 m, respectively and the water depth and flow velocity in the channel were defined as approximately 7.0 m and 2.0 m/s, respectively.

**Fig 10:**
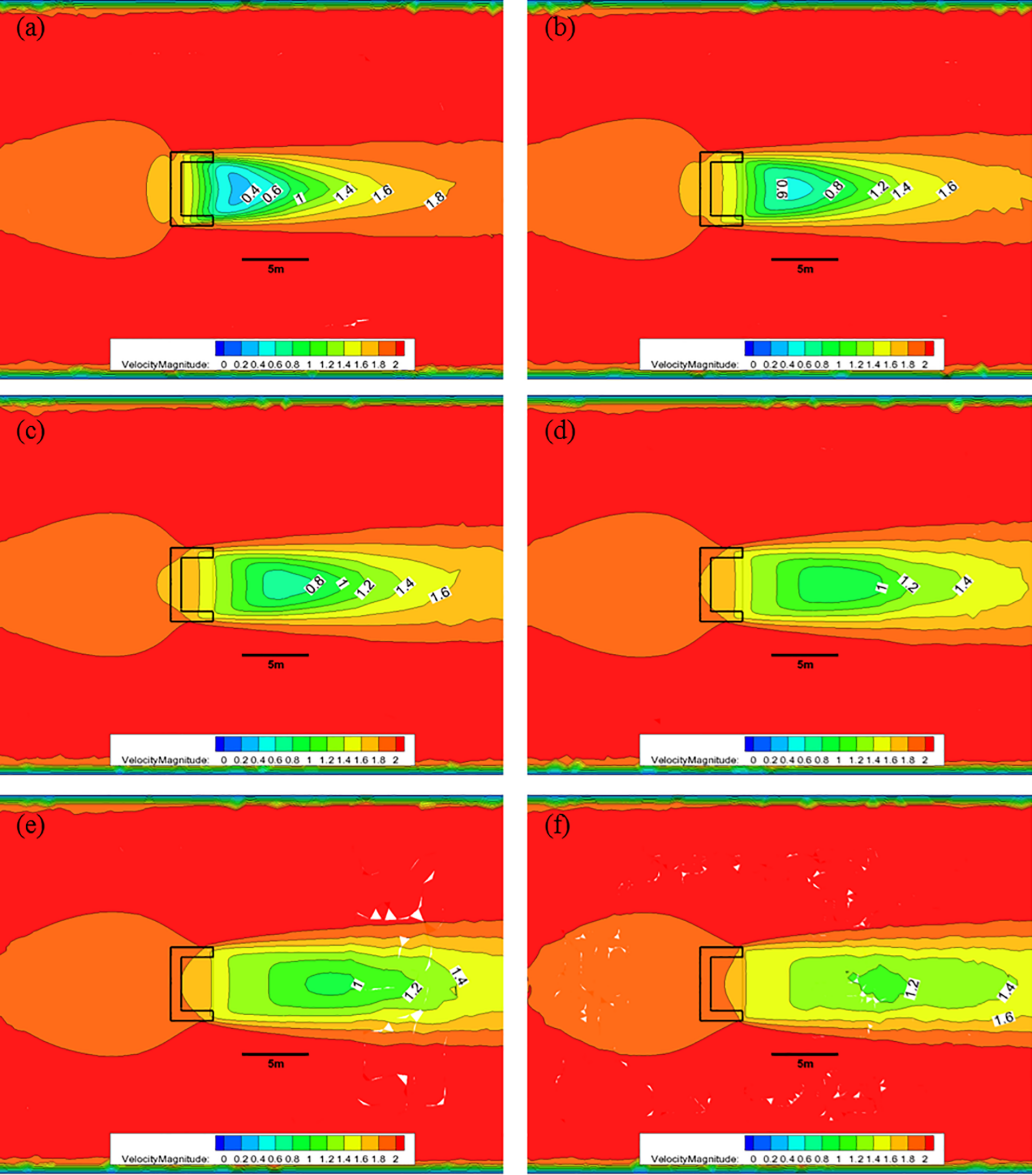
Flow of the Case 1 nose cone with 50% porosity for each water depth. (a) 0.5 m, (b) 1.0 m, (c) 1.5 m, (d) 2.0 m, (e) 2.5 m, and (f) 3.0 m.

Fig 10 shows the CFD analysis results for the nose cone of Case 1 at each water depth with 50% porosity and an initial flow velocity of 2.0 m/s. In this case, the red color represents fast flow velocities, the blue color represents slow flow velocities, and the black solid line in the middle of the figure indicates the scale, corresponding to a size of 5.0 m. Fig 10 shows that at a depth of 0.5 m with 50% porosity, the flow velocity decreases from 2.0 m/s to approximately 1.0 m/s after passing through the front flow rate reduction device; however, the length over which the velocity reduction occurs is smaller than the other cases. At a depth of 1.0 m, the flow velocity decreases to approximately 1.2 m/s over a length that is larger than 5.0 m. As the water depth decreases, the length of flow deceleration region is reduced. Furthermore, the flow velocity is greater than 1.8 m/s in most areas at a depth of 3.0 m, which is the lower portion of the flow rate reduction device.

**Fig 11:**
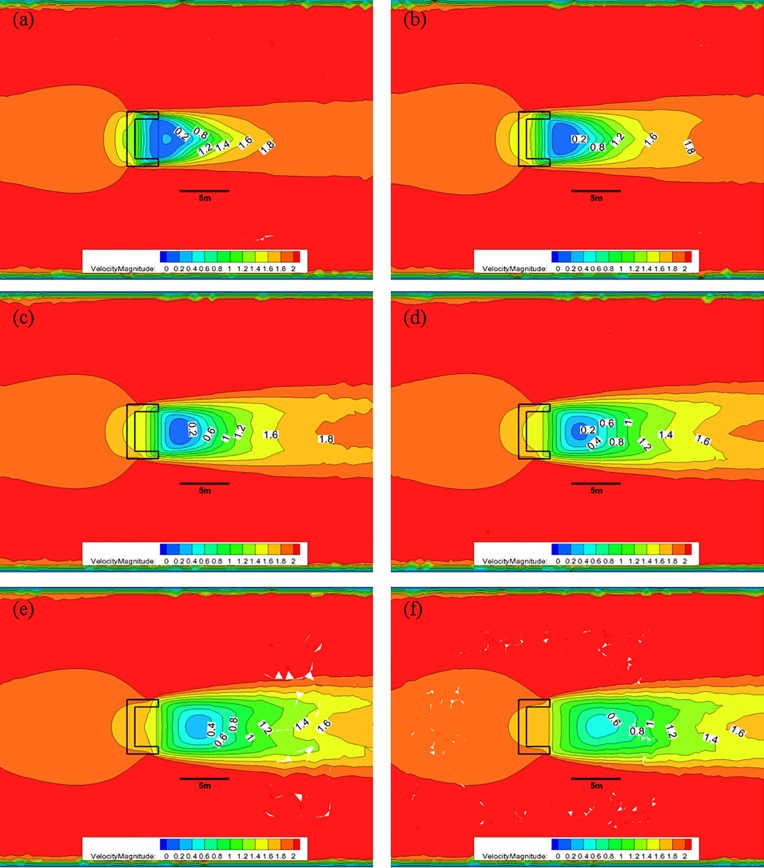
Flow of the Case 1 nose cone with 33% porosity for each water depth. (a) 0.5 m, (b) 1.0 m, (c) 1.5 m, (d) 2.0 m, (e) 2.5 m, and (f) 3.0 m.

Fig 11 shows the CFD results of the flow characteristics analysis at each depth for the nose cone of Case 1 with a porosity of was 33%. After the flow pass through the flow reduction device at a depth of 0.5 m, the flow velocity decreases from 2.0 m/s to approximately 0.2 m/s. At a depth of 1.0 m, a flow velocity of 0.6 m/s is maintained for a length of approximately 5.0 m. As the water depth decreases, the deceleration length decreased and the flow velocity is greater than 1.4 m/s in most areas at a depth of 3.0 m, which is the lower portion of the front flow reduction device.

**Fig 12:**
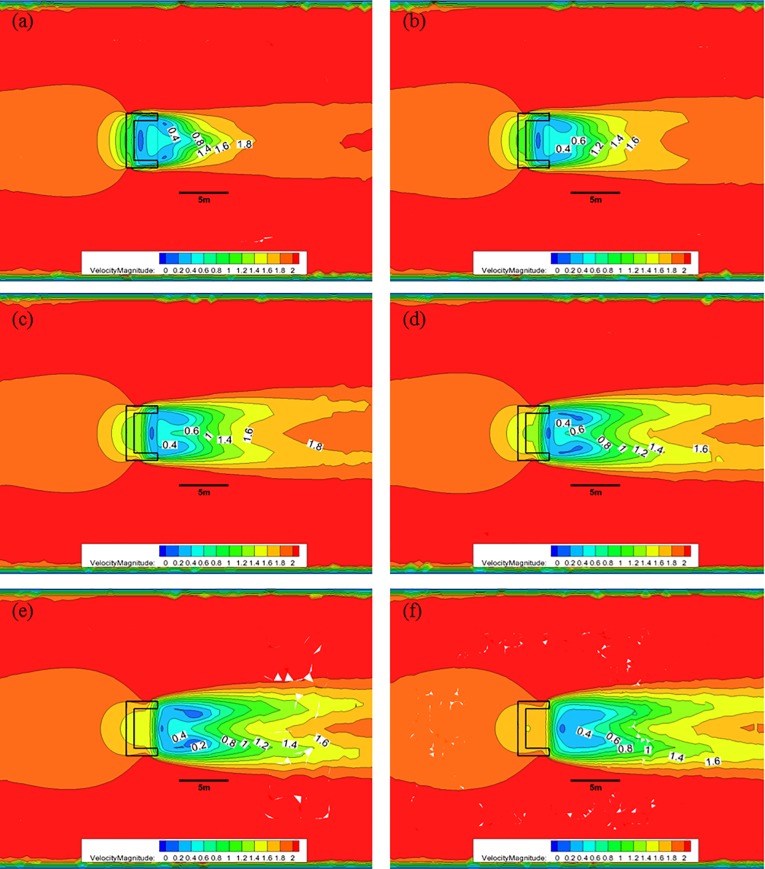
Flow of the Case 1 nose cone with 10% porosity for each water depth. (a) 0.5 m, (b) 1.0 m, (c) 1.5 m, (d) 2.0 m, (e) 2.5 m, and (f) 3.0 m.

Fig 12 shows the CFD analysis results of the flow characteristics analysis at each depth for the nose cone of Case 1 with 10% porosity. In this case, after the flow pass through the flow rate reduction device at a water depth of 0.5 m, the flow velocity decreases from 2.0 m/s to approximately 0.2 m/s. At a depth of 1.0 m, a deceleration to a flow velocity of 0.4 m/s is maintained for a length of approximately 5.0 m. As the water depth decreases, the deceleration length is reduced, and the flow velocity is greater than 1.0 m/s in most areas at a depth of 3.0 m, which is the lower portion of the flow rate reduction device.

**Fig 13:**
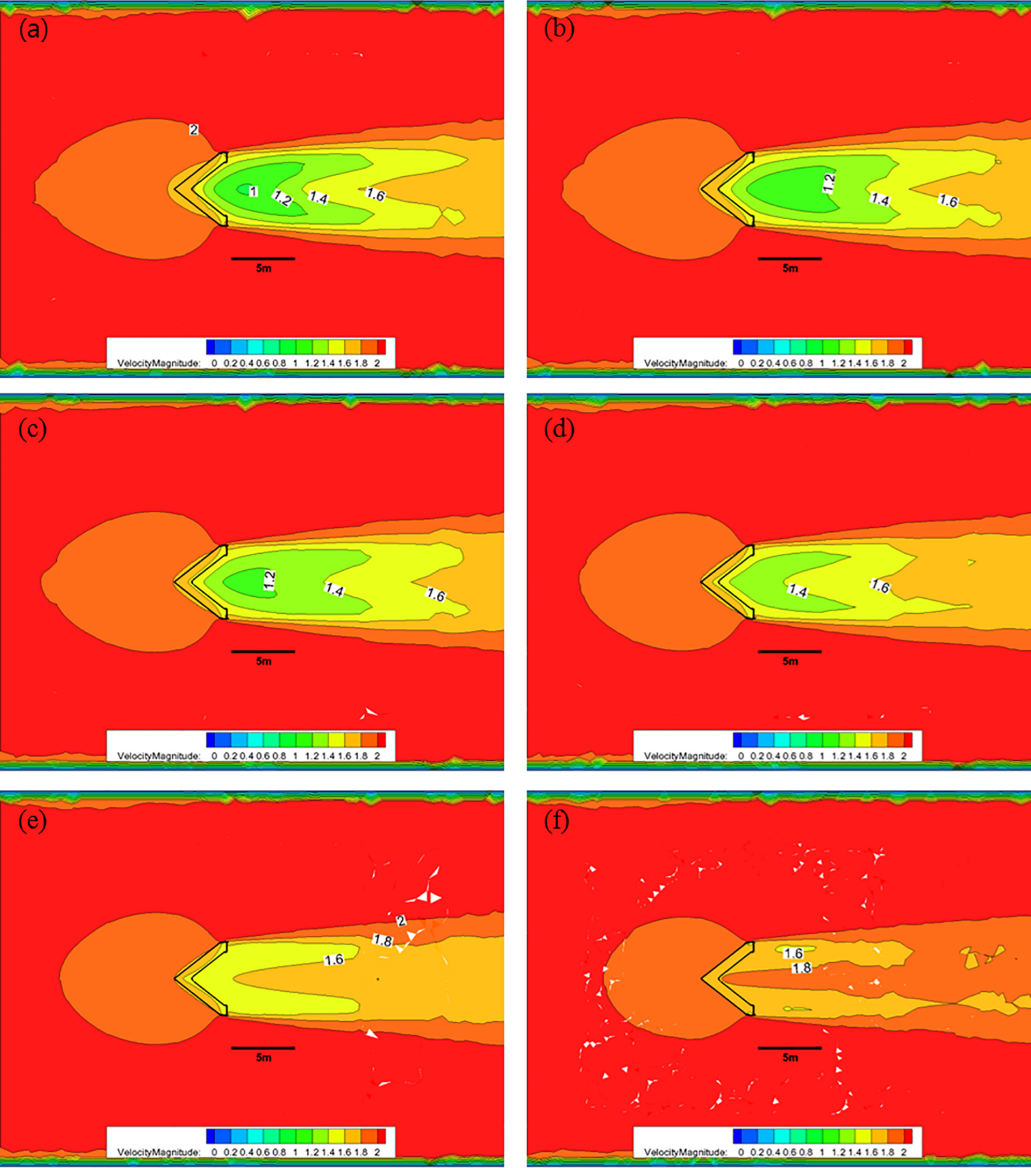
Flow of the Case 2 nose cone with 50% porosity for each water depth. (a) 0.5 m, (b) 1.0 m, (c) 1.5 m, (d) 2.0 m, (e) 2.5 m, and (f) 3.0 m.

Fig 13 shows the CFD analysis results for the nose cone of Case 2 at each water depth with 50% porosity, and an initial flow velocity of 2.0 m/s. As indicated in Fig 10, when the nose cone of Case 1 has 50% porosity, the flow velocity decreases from 2.0 m/s to approximately 0.4 m/s after passing through the nose cone at a water depth of 0.5 m; however, the length over which this flow decreases occurs is reduced. At a water depth of 1.0 m, the flow velocity decreases to approximately 0.8 m/s and is maintained for greater than 5.0 m. As the water depth decreases, the length over which the flow velocity reduction occurs is smaller, and the flow velocity is mostly maintained at a value greater than 1.2 m/s for a water depth of 3.0 m at the bottom of the nose cone.

**Fig 14:**
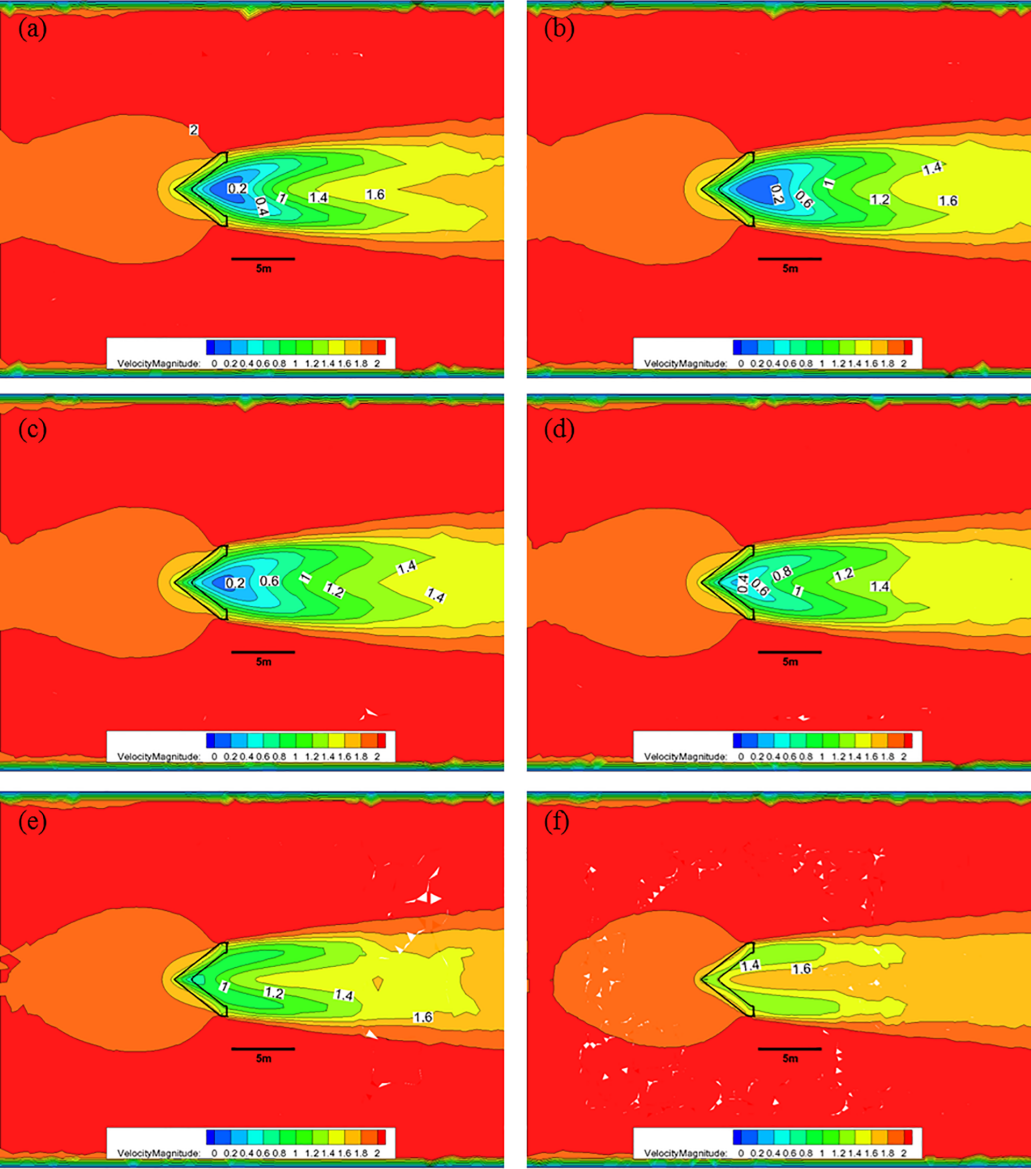
Flow of the Case 2 nose cone with 33% porosity for each water depth. (a) 0.5 m, (b) 1.0 m, (c) 1.5 m, (d) 2.0 m, (e) 2.5 m, and (f) 3.0 m.

Fig 14 shows that, when the nose cone of Case 2 has a porosity of 33%, the flow velocity decreases from 2.0 m/s to approximately after passing through the nose cone at a water depth of 0.5 m. Moreover, the length over which this flow velocity decreases occurs larger at a water depth of 1.0 m, and the flow velocity of 0.8 m/s is maintained for a distance greater than 5.0 m. As the water depth decreases, the length of the flow velocity decrease is reduced, and a flow velocity of 0.6 m/s is mostly maintained at a water depth of 3.0 m near the bottom of the nose cone.

**Fig 15:**
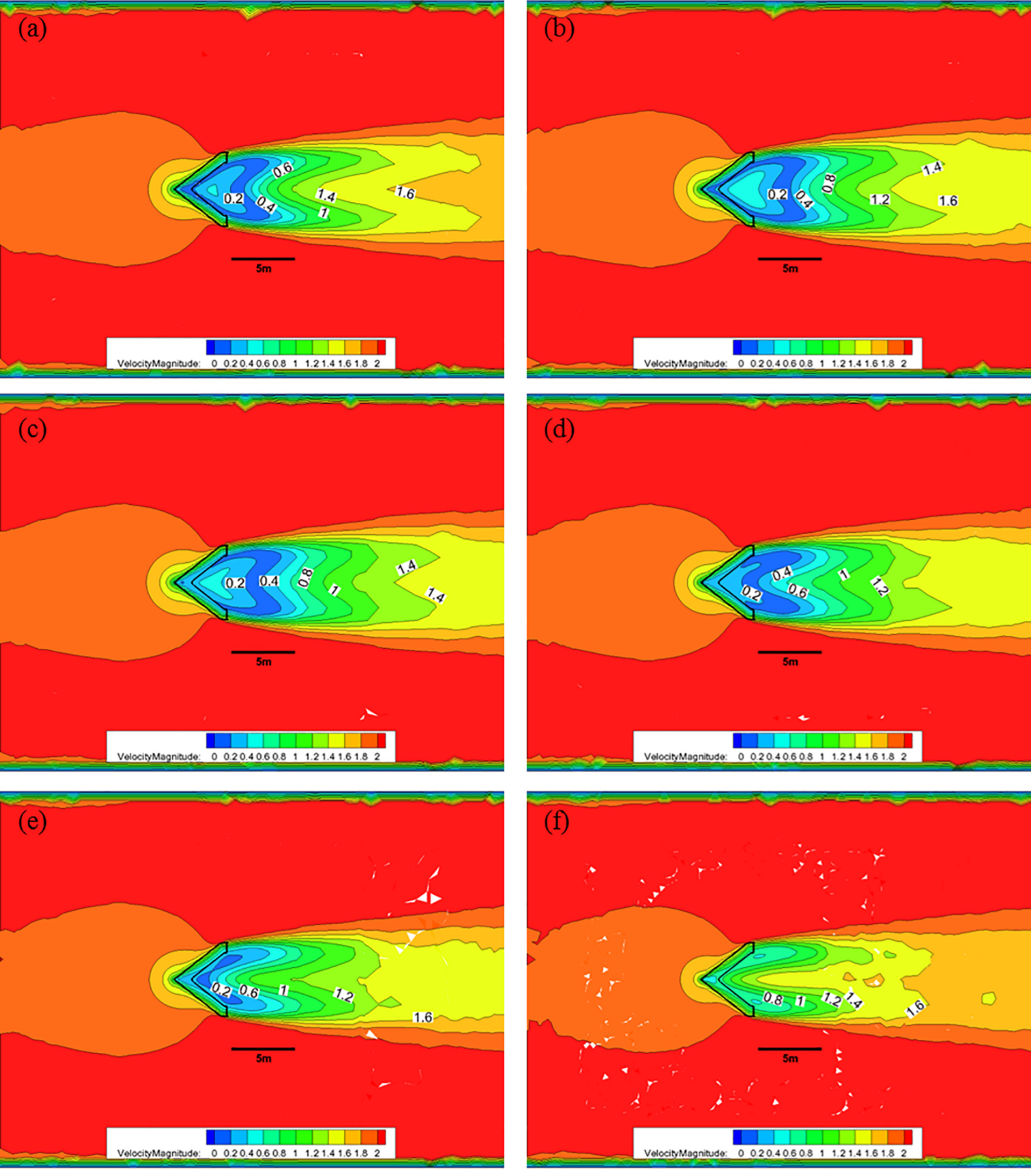
Flow of the Case 2 nose cone with 10% porosity for each water depth. (a) 0.5 m, (b) 1.0 m, (c) 1.5 m, (d) 2.0 m, (e) 2.5 m, and (f) 3.0 m.

Fig 15 shows that, when the nose cone of Case 2 has a porosity of 10%, the flow velocity decreases from 2.0 m/s to approximately 0.4 m/s after passing through the nose none at a water depth of 0.5 m. Compared with the flow velocities of the 30% and 50% porosity cases, the 10% porosity case produces a high flow velocity immediately after passing through the nose cone. This is due to the vortex that occurs in the wake of the nose cone caused by the low rates of discharge. The length over which the flow velocity decreases to approximately 0.4 m/s is widened at a water depth of 1.0 m, and the length over which the flow velocity remains less than 0.8 m/s was maintained at a distance of more than 5.0 m. As the water depth decreases, the length over which the flow velocity reduction occurs becomes smaller, and the flow velocity of 0.6 m/s is mostly maintained at a water depth of 3.0 m near the bottom of the nose cone.

As described above, the flow rate magnitude and the flow velocity reduction region after the flow passes through the nose cone differed according to the porosity. The velocity reduction region for the nose cone of Case 1 was larger and more uniform than that of the V-shaped nose cone of Case 2. Additionally, the velocity reduction for the nose cone of Case 1 according to depth was smaller than that of Case 2. In particular, the nose cone of Case 2 with 50% porosity generated a flow velocity of more than 1.0 m/s in most regions, which is considered unsuitable for fish growth. This indicates that the nose cone shape of Case 1 is more suitable as the front flow reduction device. In addition, the porosities of the nose cone should range between 10% and 33% for the fish cage system deployed in the constructed channel.

### CFD analysis of the cage system

As discussed in an earlier section, the use of CFD techniques is becoming more prevalent for assessing the flow field characteristics from a 3D perspective. A series of CFD simulations were performed with the model of the aquaculture cage system placed within the channel. Along with the cage and trapezoidal dimensions previously described, the length of the domain was set to 100 m. The free-stream flow was established as 1.31 m/s, and the flow characteristics were obtained in the *x*-*z* and *x*-*y* planes along the channel and cross-channel directions, respectively.

**Fig 16:**
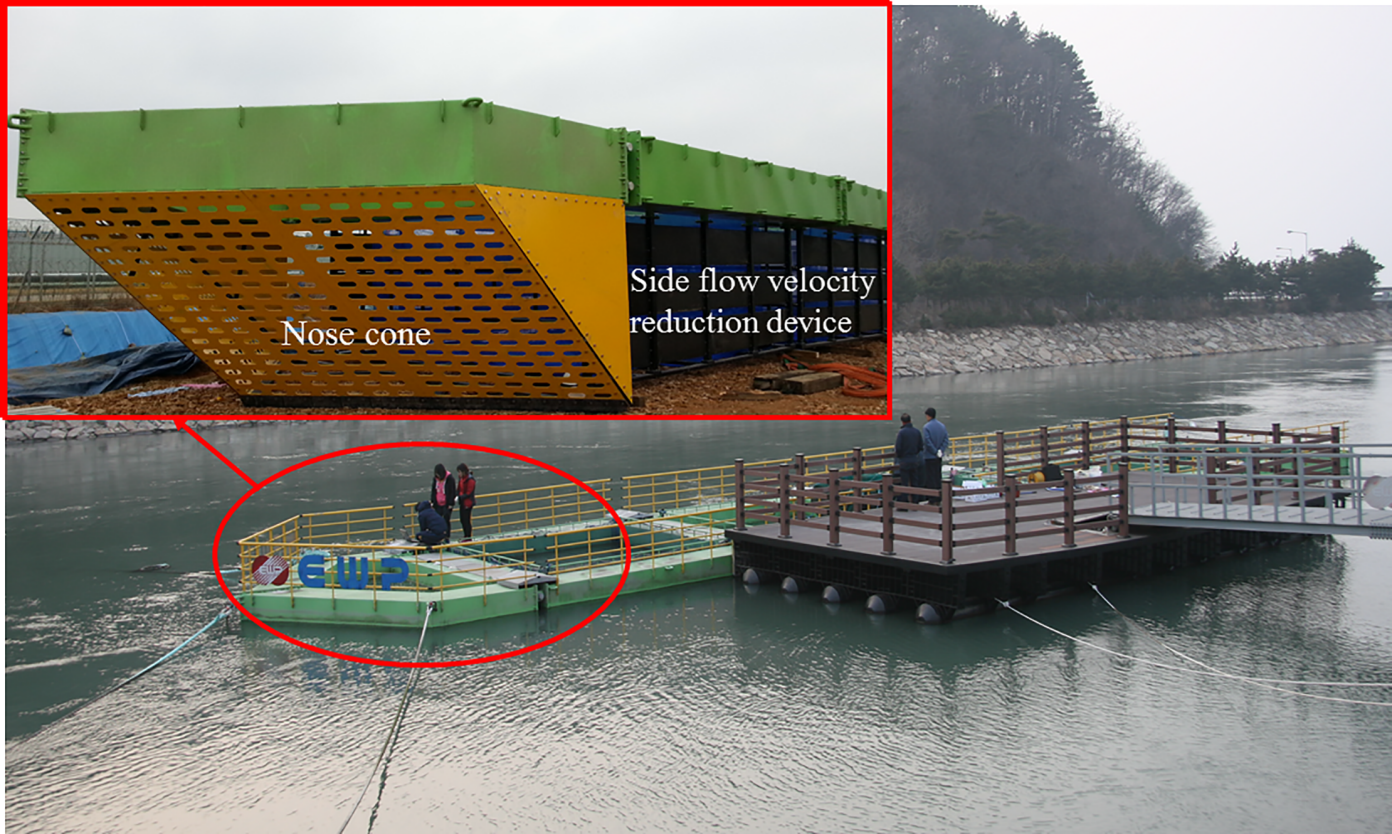
Thermal cage system with the perforated nose cone and side flow velocity reduction devices deployed in the constructed channel.

**Fig 17:**
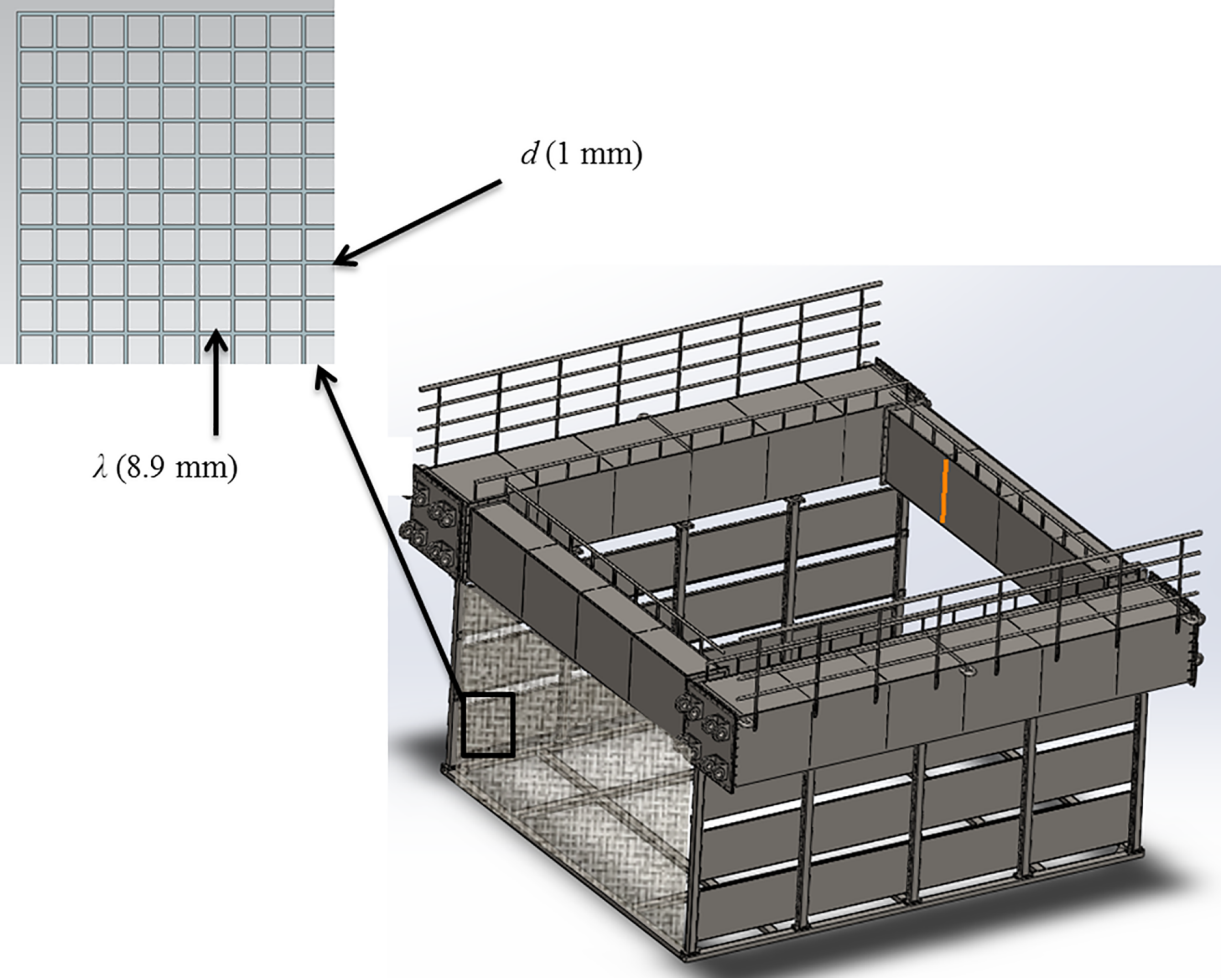
Cage frame and netting of the channel cage system.

Boundary and initial conditions. Fig 16 shows the containment net shape of the channel cage system with the nose cone of Case 1 and the side flow velocity reduction devices. As suggested in Fig 9, ***A***_***p***_ is 9.32 m^2^ and ***A***_***f***_ is 2.30 m^2^, leading to porosity of 24.7%. The thickness of the nose cone is 6.0 mm. Fig 17 shows that the mesh shape of the netting is square, and the mesh bar length (*λ*) is 8.9 mm. Meanwhile, the twine diameter is 1.0 mm, and its solidity ratio is 0.212. The size of the net is 4.4 m × 4.2 m × 2.8 m. When analyzing the flow characteristics of the net, a model using perforated plates like those in the nose cone is generated and channel cage system, as mentioned above.

**Fig 18:**
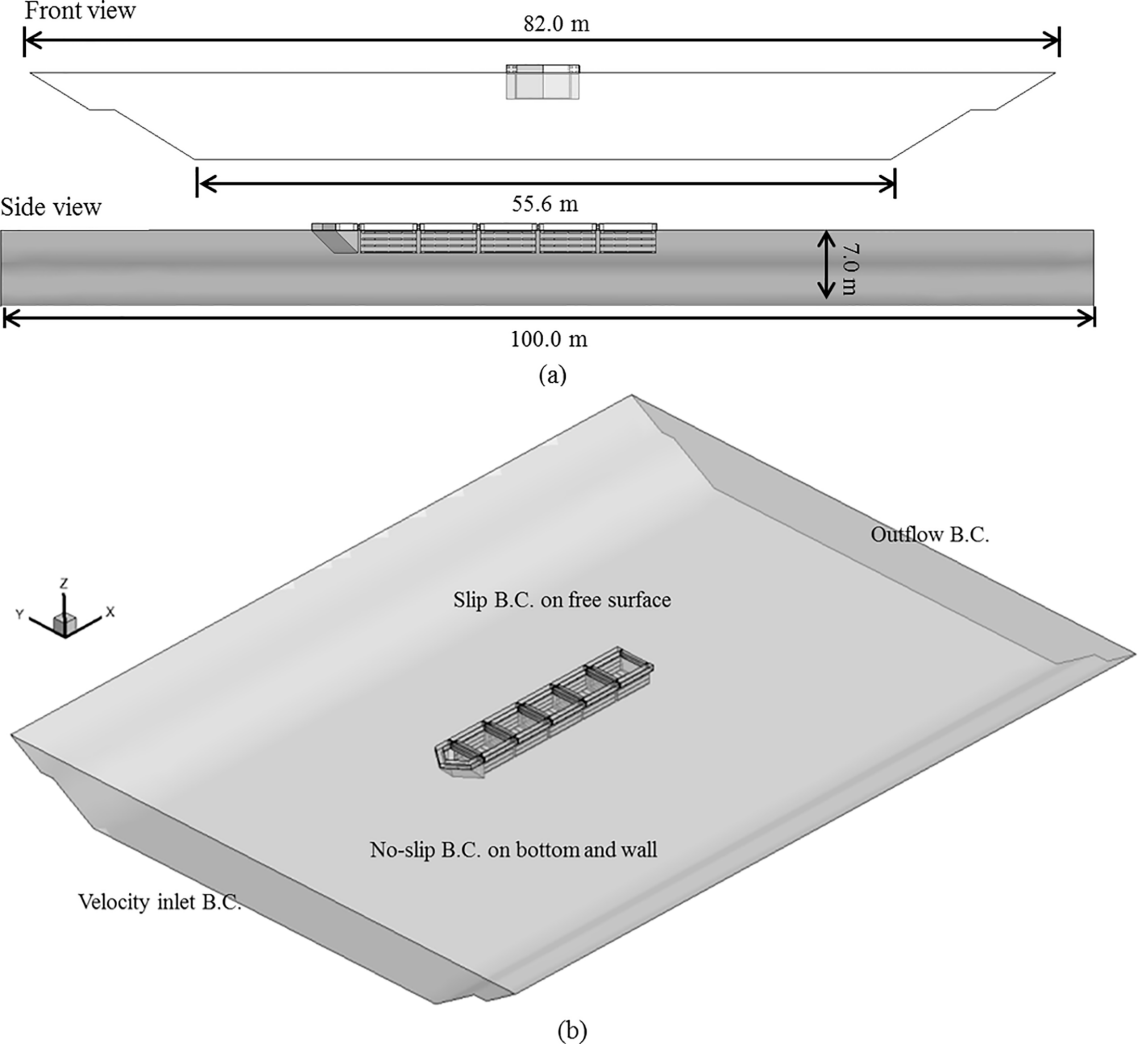
(a) Geometries of the simulation domain and (b) boundary conditions of the simulation.

**Fig 19:**
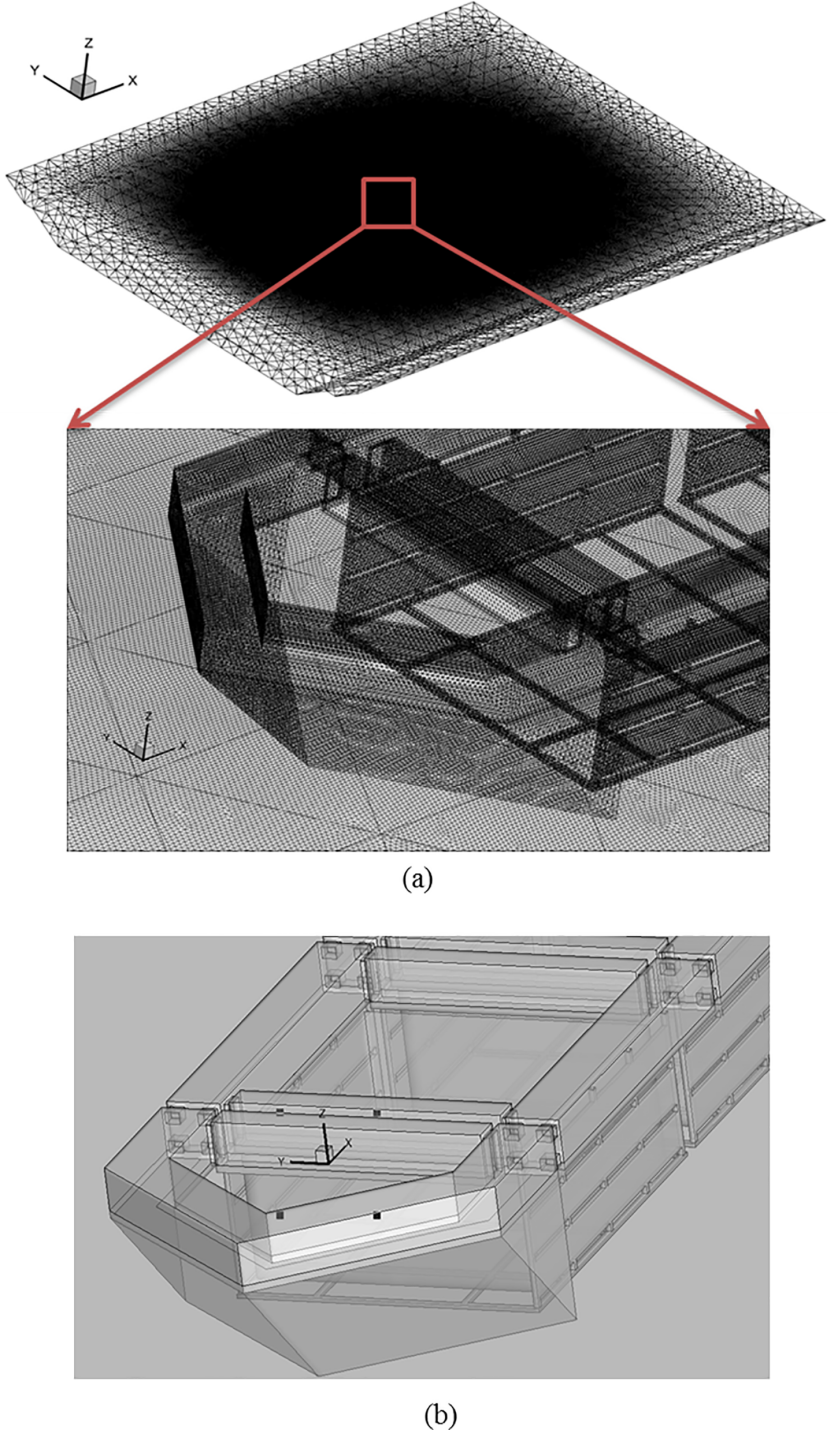
(a) Grid generation for the simulation domain and (b) coordinate system and position of origin.

The computational model domain size for each simulation was 100 m × 82 m × 7 m, as shown in Fig 18(A). The assumed boundary conditions (BCs) are described in Fig 18(B). The least absolute normalized error (LANE) approach was applied to determine the friction coefficient of the netting, as described by the viscous loss term of Eq (3). The values for the porous media friction coefficients, as described in Eq (7), were set to the values reported by Patursson et al. [10]: *D*_*n*_ = 51,730 m^-2^, *D*_*t*_ = 26,379 m^-2^, *C*_*n*_ = 5.0980 m^-1^, and *C*_*t*_ = 1.6984 m^-1^. The computational domain was discretized into 6,892,134 tetrahedral cells with 14,485,234 faces as shown in Fig 19(A). The tetrahedral mesh type was used in the analysis. The initial velocity of the flow was set to 1.31 m/s at the beginning of the analysis. Fig 19(B) shows the coordinates and starting point of the CFD calculation area. Fig 19(B) establishes the stream-wise direction, the span-wise direction (at a right angle to the flow), and the vertical direction as the *x*, *y*, and *z* coordinate axes, respectively. Furthermore, the starting point is located in the *z* direction at a depth of 10 cm beneath the water surface. Therefore, the *z* coordinate of the water surface is 10 cm. The starting point in the *x* direction is location at which the nose cone ends, and the starting point in the *y* direction is located the center of the channel cage system.

**Fig 20:**
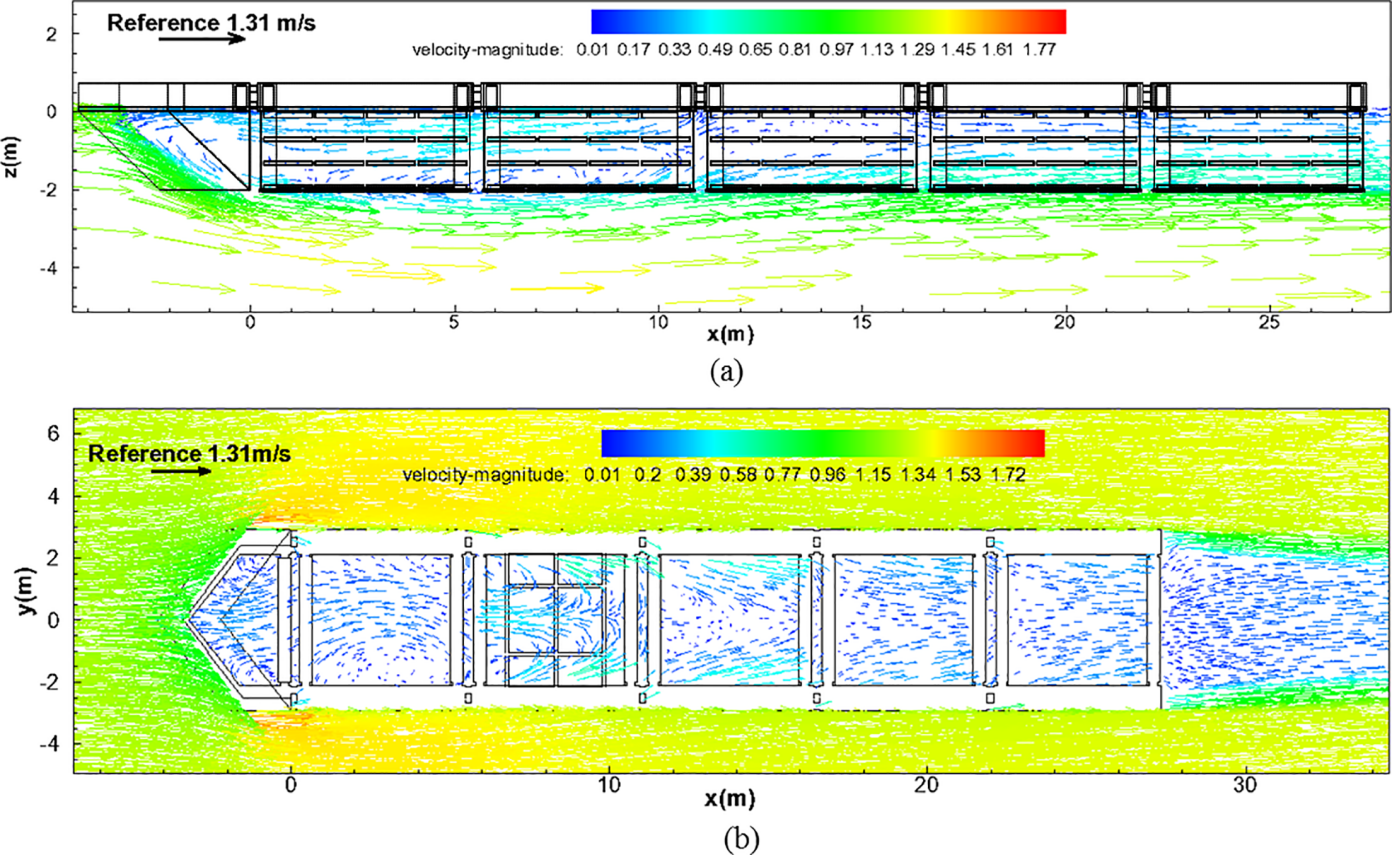
Flow vector of the channel cage system from the CFD analysis. (a) *x*–*z* plane and (b) *x*–*y* plane.

Flow velocity vector field. Fig 20(A) shows the velocity vector field of the *x*–*z* plane, which presents the flow characteristics within the channel cage system of the installed nose cone (see S1 Movie). In this figure, the red vector color indicates high velocities, whereas the blue vector color indicates low velocities. For the specific velocities, refer to the legend included in Fig 20(A). The top left of Fig 20(A) presents a standard vector that corresponds to the initial flow velocity of 1.31 m/s. The analysis of the *x*-*z* plane flow vector shape shows that the flow velocity at the bottom of the channel increases as the fluid that could not pass through the nose cone flows into the bottom of the channel. Owing to this phenomenon, a vortex occurs near the range of *x* = 4.0 m–8.0 m and at point of *z* = -2.0 m, which is the location where the low-velocity fluid of the channel cage system and the high-velocity fluid from the bottom of the channel meet. Note that, in Fig 20(A), the flow vectors appear in a comparatively constant direction, similar to the direction of the entrance flow, after the location of approximately *x* = 12 m. An apparent backflow 6 m to 10 m occurs in the second cage due to the complicated 3D flow created by the vortex when the fluid passing through the nose cone flows downstream of the cage.

As shown in Fig 20(B), the analysis of the *x*-*y* plane flow vector shape demonstrates that the flow velocity at the sides of the channel cage system increases, as the fluid that could not pass through the nose cone flows into both the bottom and the sides of the channel cage system (see S2 Movie). However, unlike Fig 20(A), a distinct vortex does not appear in this case. This is because the side flow velocity reduction system is installed in the channel cage system, and, thus, there is reduced region where the low-velocity fluid in the channel cage system meets the high-velocity flow at the bottom of the channel. In Fig 20(B), the flow vectors appear in a comparatively constant direction, similar to the direction of the entrance flow, after the location at approximately *x* = 12 m.

**Fig 21:**
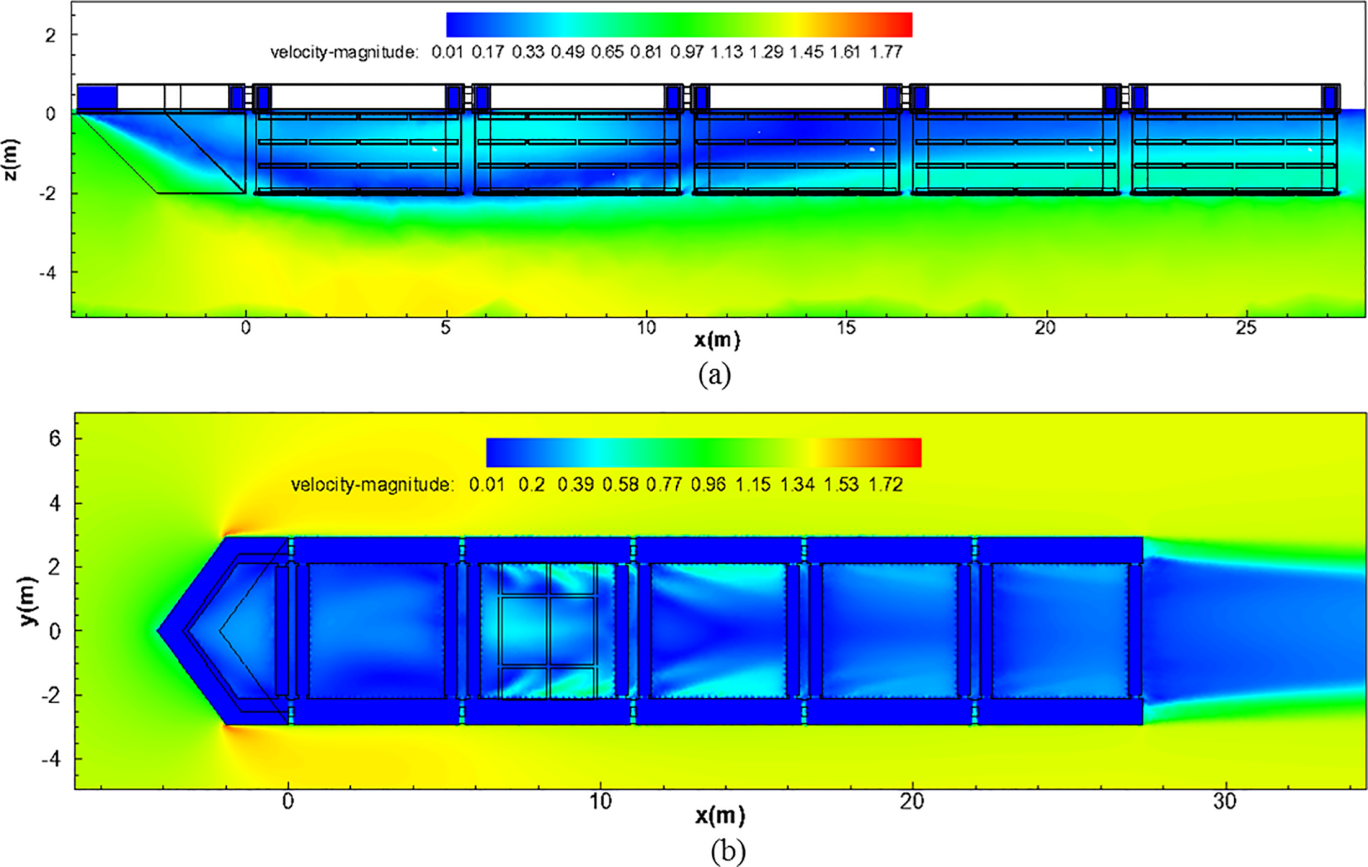
Flow velocity magnitude of the channel cage system from the CFD analysis. (a) *x*–*z* plane and (b) *x*–*y* plane.

Flow velocity distribution. To determine the flow characteristics within the channel cage system with the installed nose cone, Fig 21(A) shows the velocity magnitude contour plot of the *x*–*z* plane. In the scale presented in Fig 21, the red color indicates high velocities, whereas the blue color indicates low velocities (see S3 Movie). Considering the flow velocity distributions of the *x*–*z* plane, we believe that the nose cone functions appropriately. The flow velocities of the first net cage are lower compared with those of the initial stage. Owing to the effects of the vortex mentioned previously, an area of low flow velocity is presented, and the velocity increases again after *x* = 17 m.

Fig 21(B) shows the *x*-*y* plane velocity magnitude contour plot of the channel cage system (see S4 Movie). The flow velocity distributions of the *x*–*y* plane show that a distinct vortex form does not appear; instead, comparatively constant flow velocity distributions are revealed, unlike the *x*–*z* plane shown in Fig 21(A). This is because the side flow velocity reduction system is installed at the sides of the channel cage system, and, thus, there is a reduced region where the low-velocity fluid in the channel cage system directly meets the high-velocity fluid at the bottom of the drainage channel.

**Fig 22:**
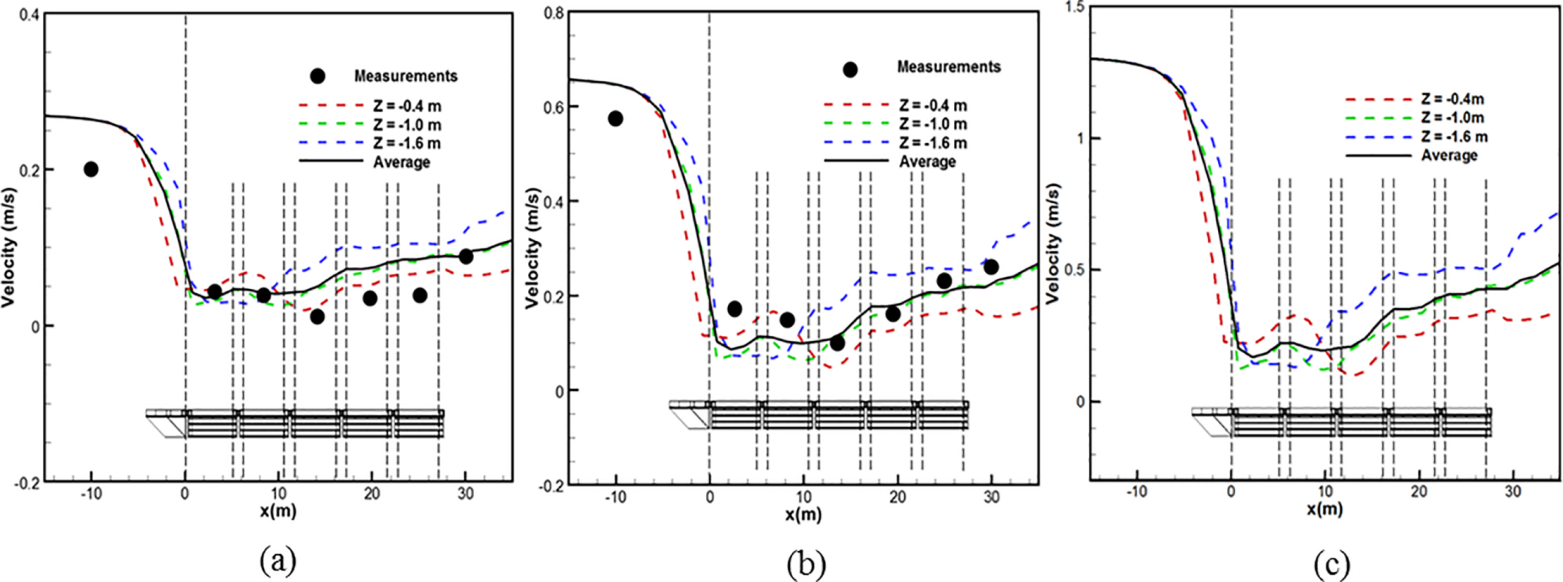
Summary of the flow velocity results generated by CFD analysis and measurements for three initial velocities. (a) 0.27 m/s, (b) 0.66 m/s and (c) 1.31 m/s.

Average flow velocity distributions. The objective of examining the average flow velocity distributions is to gain further understanding of the flow reduction through the perforated nose cone of the cage system in the constructed channel. Flow velocity measurements in the channel and inside the cage system were made to assess the CFD results. Velocity measurements at *x*-, *y*-, and *z*-distance of approximately 2.7 m, 2.5 m, and 1.0 m were obtained with a Nortek Aquadopp Acoustic Doppler Velocimeter (ADV) in May and August 2014. Measurements were obtained at a frequency of 23 Hz for 2 min at each point (*n* = 2760). Fig 22 shows a summary of the flow velocity results generated by the CFD analysis and measurements. This shows the results for the initial flow velocities of 0.27 m/s, 0.66 m/s and 1.31 m/s, respectively. The dotted lines indicate the average flow velocities at each height in the direction of *z*. The solid lines indicate the average values and the summation of the values and the average flow velocities of the heights concerned. Fig 22(A) and 22(B) shows that the CFD results are in good agreement with the measured results. As shown in Fig 22, the velocity at the free-stream level (a position 14 m in front of the cage) experiences a large reduction to a velocity of approximately 0.1 m/s as the flow passes through the nose cone. As the flow passes around, under, and through the cage system, the flow velocity re-establishes with velocities approaching 0.2 m/s at an *x*-distance of 25 m. In the case of Fig 22(C), where the initial flow velocity is 1.31 m/s, the flow decreases from approximately 1.3 m/s to 0.25 m/s as it passes through the nose cone (the range of approximately -4.0 m–0.0 m). The flow velocity remained within 0.3 m/s to the point where it passes through the second net of the channel cage system (at approximately 14 m). The velocity gradually increases as the flow approaches the fifth net of the channel cage system (at approximately 25 m), where the velocity reaches 0.4 m/s.

## System loads analysis

In addition to analyzing the flow characteristics of the channel cage system, an additional focus of this study was to determine the potential structural stresses on the fish cage system as a result of the drag loading from the local flow velocities. The CFD analysis also provided the average velocity values, which are useful for analyzing the drag loading on the cage system. Building upon this work, this study aimed to implement the same CAD models use for the finite element analysis (FEA) software to assess the critical component stresses. First, the drag loads were calculated from current velocity from the CFD simulations and the wind speed, and then the drag loads were applied in the FEA. Additional load cases were also applied to represent the deployment and recovery operations. The stresses were examined on both the nose cone and the containment volume sections.

The system loads were considered for the components that are both in and above the water. In the water, the nose cone portion of the system was subjected to the free-stream velocity of 2.0 m/s. A reduced current velocity of 0.75 m/s was applied to the individual fish net cages. The wind speed on the above-water components was estimated as 14.0 m/s, considering the power plant location of Dangjin, South Korea. In this analysis, the drag loads on both the nose cone and the containment module (i. e. the fish cage) were estimated, assuming the individual structural components were subjected to steady flow, according to Eq (9).

$$F_{drag}=\frac{1}{2}\rho C_{d}AV^{2},$$ (9)

Where *ρ* is the fluid mass density (1025 kg/m^3^ for seawater) and *A*, *C*_*d*_, and *V* are the projected area, drag coefficient, and flow velocity for each individual component, respectively. This approach was also used to calculate the drag on each of the fish cages.

### Overview

The structural analysis was performed using the commercially available CAE software package, SolidWorks, as described in Planchard and Planchard [20]. The finite element simulations of the frame (the load-bearing component) were conducted to investigate the structural integrity, and establish the service load limits of the frame. Basic carbon steel was the material used in all of the calculations. The carbon steel was assumed to be linear elastic and isotropic with a modulus of elasticity of 210 GPa and a Poisson’s ratio of 0.28. The distribution of the equivalent stresses (von Mises) was analyzed to determine the locations of the stress concentrators. Note that the data from the finite element analysis can sometimes predict excessively high localized stresses, which are generally located at sharp corners in the component geometry. Therefore, the single highest finite element stress that is observed in the analysis is not necessarily representative of the maximum stresses of the actual components. Thus, we report the volume ratio of the elements (0.1% of all elements by volume) with the highest stresses, which can be used in the set of design criteria. For example, the design material should be chosen such that 99.9% of the elements (by volume) will not exceed the yield stress (*σ*_99.9%_).

#### Nose cone

**Fig 23:**
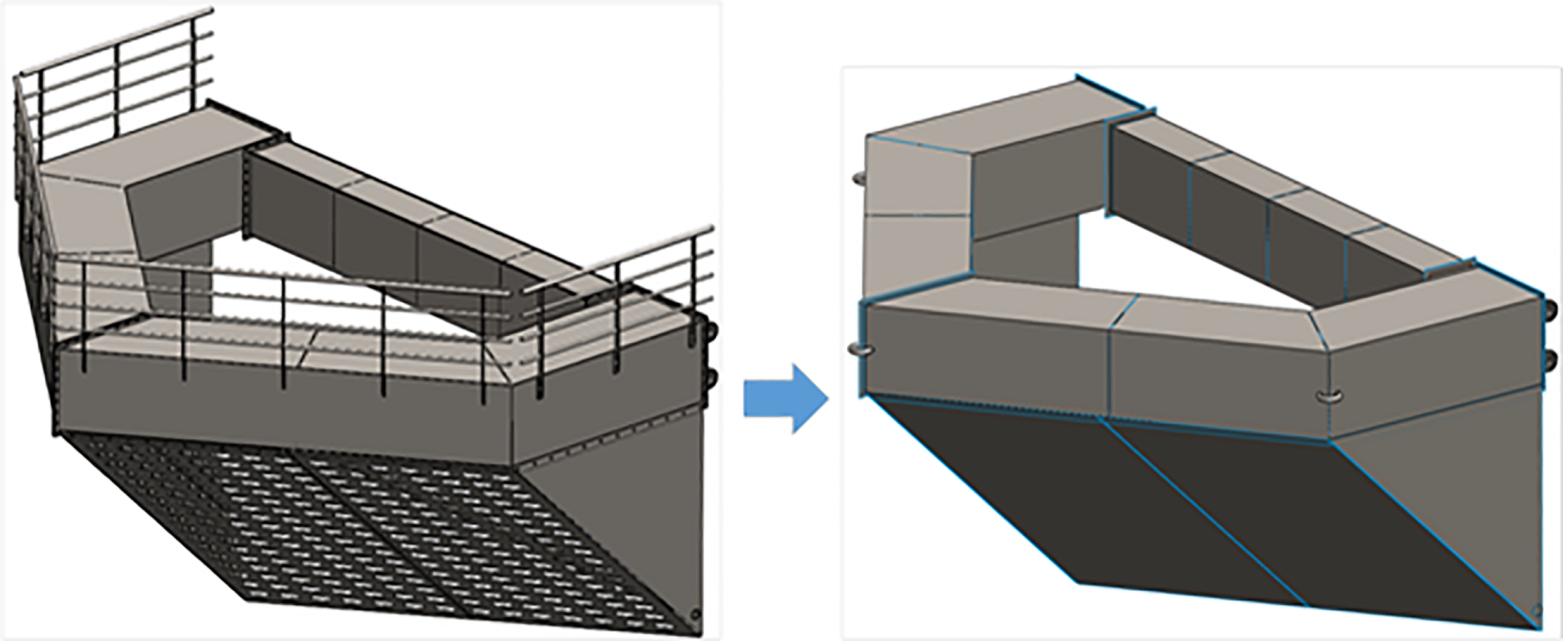
Initial and simplified geometries of the nose cone used in the structural analysis.

**Fig 24:**
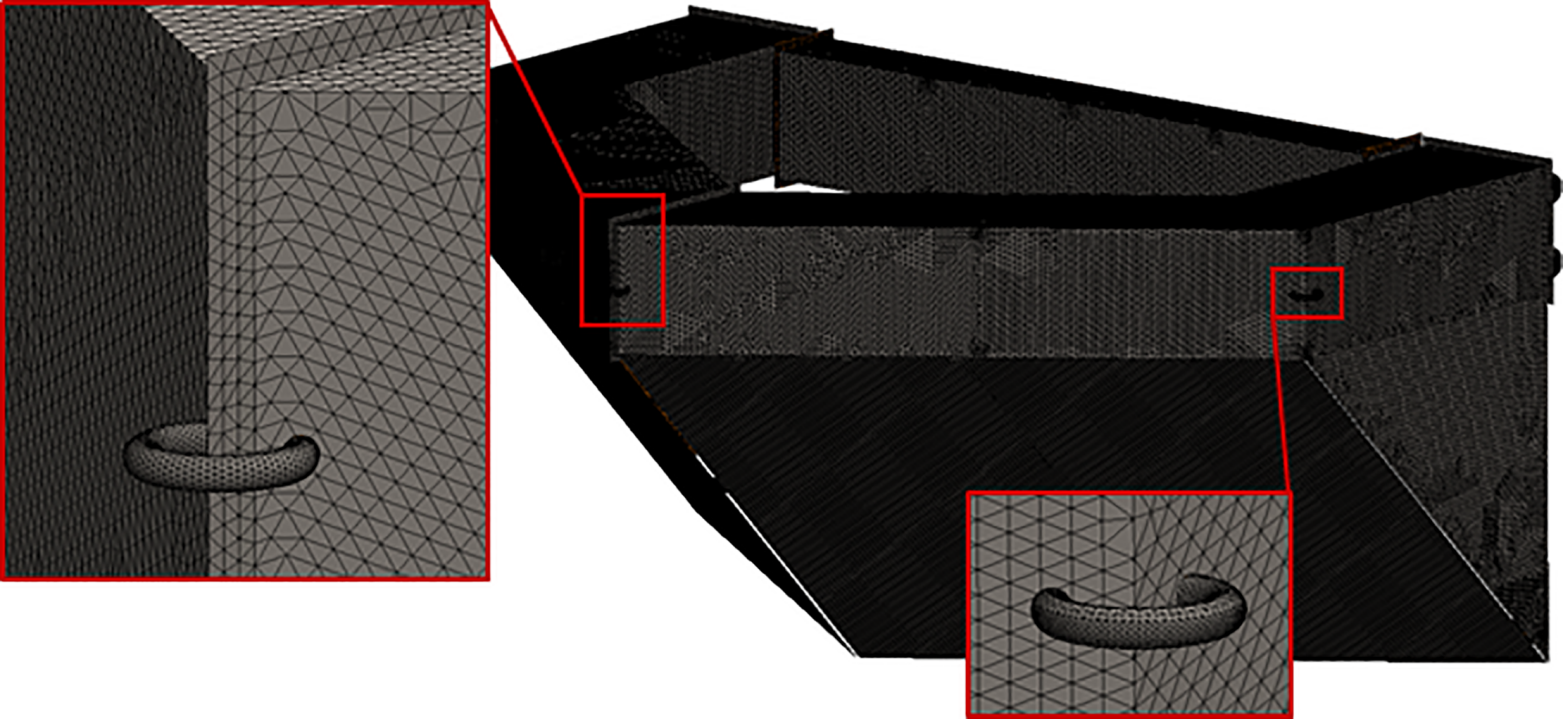
Finite element mesh of the nose cone.

Finite element analysis was used to model the mechanical response due to the dynamic loads of the nose cone using the quasi-static load cases. The geometry was designed in the CAD software, SolidWorks, and the analysis was performed using the software simulation module. Prior to the finite element analysis, the geometry of the nose cone was simplified to remove the features that were not considered to be significant for the structural response analysis, as shown in Fig 23. The geometric shapes from Fig 23 were then used to obtain the computation mesh consisting of 0.3 million second-order isoparametric finite elements, as shown in Fig 24. The elements were generated having an average size of 30.0 mm with a curvature-dependent refinement option that included 16 divisions per full circle and with a minimum element size of 5.0 mm. All of the geometrical features were meshed using quadrilateral shell elements, with the exception of the hinges and mooring attachment U-hooks, which were meshed with tetrahedrons.

The carbon steel material properties were assigned using an isotropic linear elastic constitutive model with a Young’s modulus of 205 GPa, Poisson’s ratio of 0.29, and von Mises yielding criterion. The nose cone was modeled as a multi-body system with 39 separate objects. The bonded contact option (simulating that the objects are ideally glued together) with compatible mesh (the nodes of the objects are shared) was used. Direct contact between the parts (the no penetration condition for touching faces) was not modeled.

**Fig 25:**
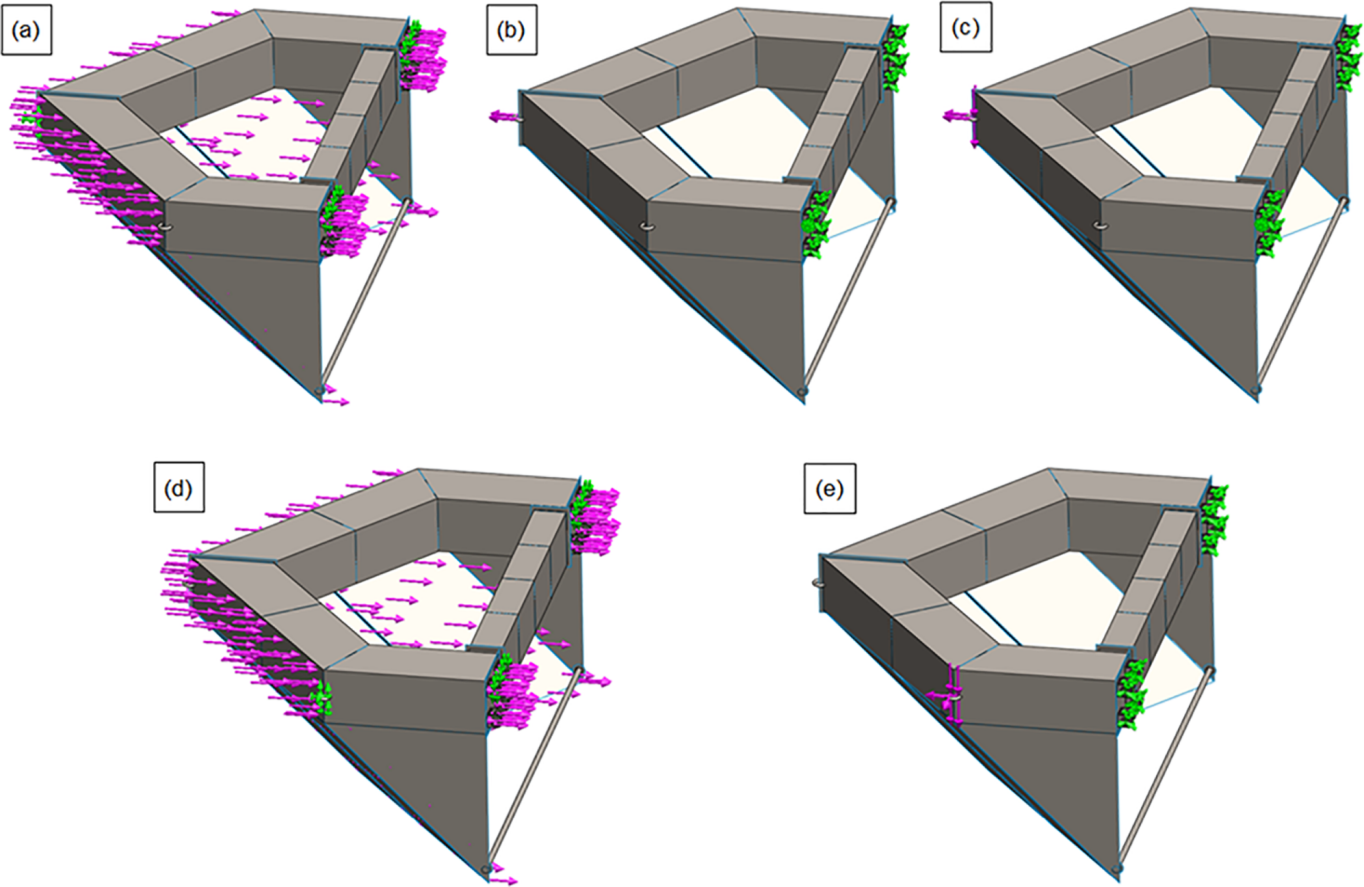
Boundary conditions of the nose cone for load cases (a)–(e).

Five load cases on the nose cone were considered, which are identified below as NC-(a) through NC-(e), and are shown in Fig 25 with BCs that replicate several loading scenarios.

NC–(a): The system was fixed at the central U-hook, simulating the constraint produced by an attached mooring line. The surfaces of the top four hinges were constrained to horizontal motion only. A horizontal load of 6.9 kN was uniformly applied to the eight hinges, simulating the drag resistance of the fish cages. A horizontal force of 5.1 kN was applied to the surfaces of the flow reduction plates, simulating the drag force acting on these panels generated by 2.0 m/s of flow. A horizontal force of 6.61 kN was applied to the frontal surfaces of the floaters, simulating the drag force acting with 2.0 m/s of flow.

NC–(b): The system was fixed at the eight hinges (the hinge BC), simulating the hinge connections of the fish cages. A horizontal total force of 18.6 kN was applied to the central U-hook, simulating the total acting force on the system, given the conditions that the central mooring counteracted the action of all of the drag forces.

NC–(c): This case has the same loading conditions as case NC–(b); however, instead of the horizontal force of 18.6 kN, a horizontal component and a vertical components of 13.15 kN were applied, simulating the tension of a mooring line that is oriented at an angle of 45° to an attachment located on the side of the channel.

NC–(d): This case has the same loading conditions as case NC–(a); however, instead of the central U-hook, one of the side U-hooks was fixed to simulate the constraint of a lateral mooring line.

NC–(e): This case has the same loading conditions as case NC–(c); however, three force components of 10.74 kN were applied to the surface of one of the side U-hooks to simulate the tension of a lateral mooring line.

### Cage frame

**Fig 26:**
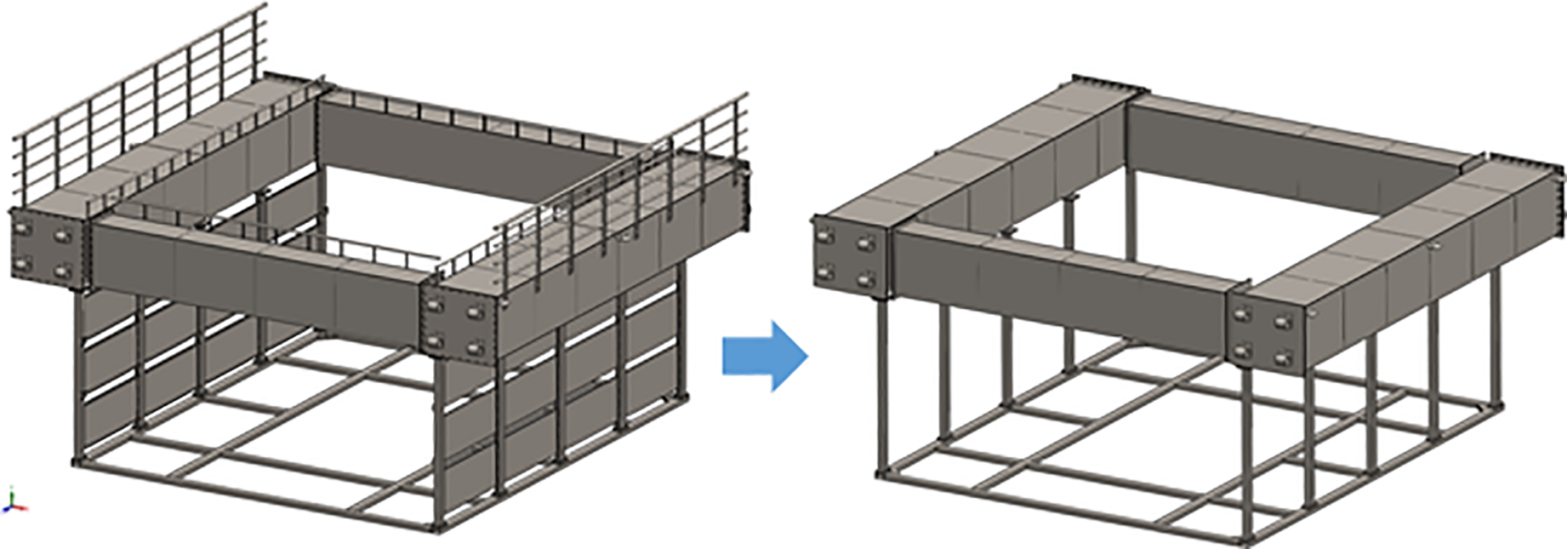
Initial and simplified geometry of the cage frame used in the structural analysis.

**Fig 27:**
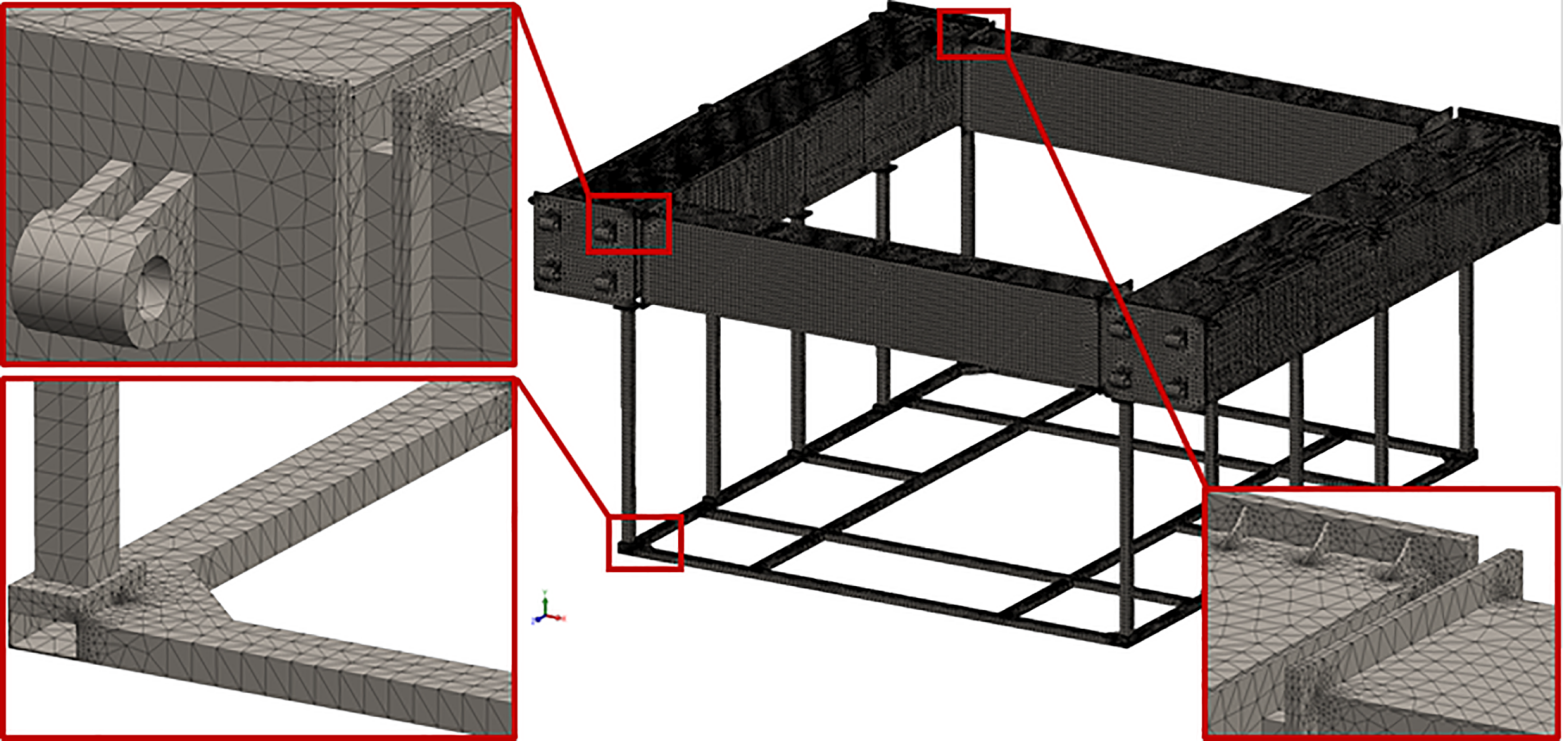
Finite element mesh of the cage frame.

In addition to the structural analysis of nose cone, finite element analysis was also used to model the mechanical response of the dynamic loads to the cage frame using the quasi-static load cases and SolidWorks. Prior to conducting the finite element analysis, the frame geometry was simplified, as shown in Fig 26 to remove the features that were not significant to the structural response analysis. The mesh shown in Fig 27 consisted of 0.63 million second-order isoparametric tetrahedrons and was generated with an average element size of 35.0 mm and with the automatic transition option of the meshing module.

The carbon steel material properties were assigned using an isotropic linear elastic constitutive model with a Young’s modulus of 205 GPa, Poisson’s ratio of 0.29, and von Mises yielding criterion. The cage frame was modeled as a multi-body system containing 23 separate objects. The bonded contact option (simulating the objects were ideally glued together) with compatible mesh (the nodes of the objects are shared) was used. Direct contact between the parts (the no penetration condition for touching faces) was not modeled.

**Fig 28:**
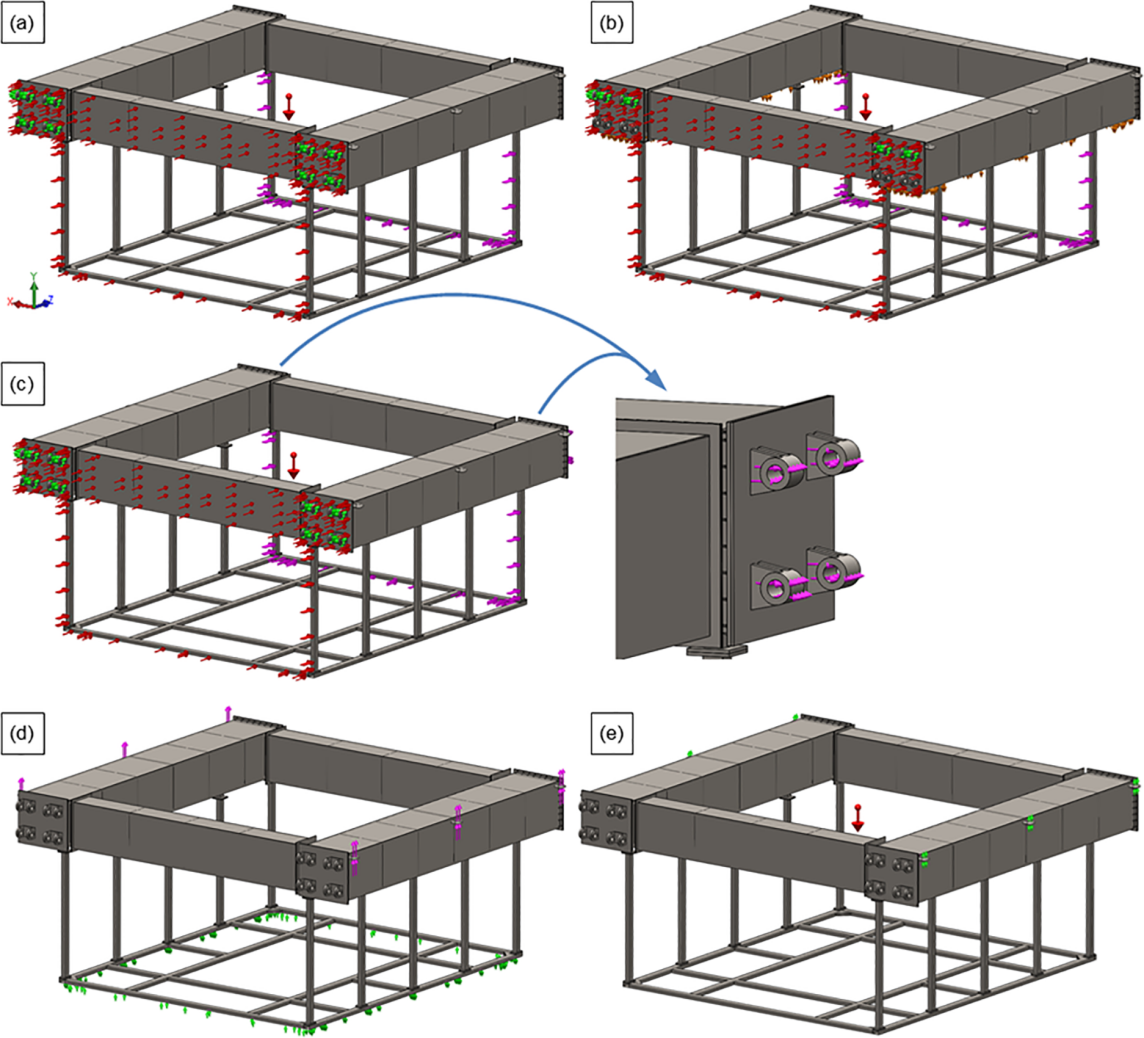
Pictorial representation of boundary conditions for frame load cases (a)–(e).

Five load cases for the cage frame, identified as FC–(a) through FC–(e), were considered, each with specific BCs. Load cases FC–(a), (b), and (c) simulate the service conditions in the water under a constant unidirectional current, whereas load cases FC–(e) and (f) simulate the assembly and transportation loads. The details of the BCs are listed below and correspond to Fig 28(A)–28(E).

FC–(a): The intent of this load case was to isolate the stresses on the hinges. The frame was fixed at the eight hinges at the front of the cage, simulating the hinge connections to the other cages. A vertical load of 40 kN was applied as a uniformly distributed body force (gravity load) to all components for simulating the load generated by the cage weight. A uniform force of 2.54 kN was applied to the front surfaces of the cage. The drag force acting on the attached netting was considered to be uniformly distributed horizontal force (a total of 1.46 kN) that was applied to the front and back supports surfaces of the cage.

FC–(b): For further examination of the hinge stresses, the loads were applied in a similar manner to those of load case FC–(a); however, in this case, the frame was fixed at the four top hinges at the front of the cage. Moreover, the bottom surfaces of the side floaters were constrained to move only in the horizontal direction.

FC–(c): The goal of this load case was to isolate the stresses on the hinges with the other four containment modules attached. In this situation, the frame was fixed and loaded in the same manner as load case FC–(a), but with an additional horizontal load (a total of 2.92 kN) was included that pulled on the eight hinges at the back of the structure. The intent was to simulate the drag force of the net panels attached to the other four structures. These additional drag values assumed that each additional containment system had only one net panel between them, due to shadowing effects.

FC–(d): To isolate the stresses on the six lifting brackets, the bottom surfaces of the frame were constrained to move only in the horizontal direction. A vertical force of 40 kN, which is equal to the weight of the system was applied to the surfaces of the six lifting brackets.

FC–(e): The stresses on the frame were examined as a part of this load case. For these simulations, the surfaces of the six lifting brackets were constrained to horizontal movement only. A vertical load of 40 kN was applied as a uniformly distributed body force (gravity load) to all of the components, which simulated the cage weight.

### Cage system load analysis

The component intended for the front and rear net panels was generated, assuming a projected area value of 1.8 m^2^ (a solidity ratio of 0.21 with an outline area of 8.5 m^2^) with *C*_*d*_ = 1.4, as described by Tsukrov [21], resulting in a value of 1.46 kN. Note that the analysis was performed without the preceding nose cone structure. This total net drag force was then applied to multiple attachment locations along the frame. An additional drag load was also assumed to act on the front face of the structure supporting the flotation element (without the nose cone). With the same mass density and water velocity values, Eq (9) was used with a projected area of 4.4 m^2^ and a *C*_*d*_ value of 2.0, as described by Tsukrov [21], such that the total force on these panels was calculated to be 2.54 kN. It should be noted that the skin friction of the components parallel to the flow was assumed to have a negligible influence on the total drag force. To simulate the mechanical response to the drag loads of the first fish cage (the one next to the nose cone component), we also considered the drag loads acting on the downstream fish cages. A force of 2.92 kN was applied to the attachment hinges, which was equivalent to the total drag acting on the four downstream net panels.

For the nose cone, the drag force was calculated by considering three distinct elements: flotation elements, reduction plates, and handrails. The flotation elements were assumed to be half-submerged, each portion having a frontal area of 1.52 m^2^. Assuming a drag coefficient of 2.0, we found the drag loads corresponding the water and wind velocities on the flotation element to be 6.23 kN and 0.38 kN, respectively. The drag on the reduction component of the nose cone was also calculated with Eq (9). Assuming a drag coefficient of 1.8 and a fully-submerged frontal of 1.38 m^2^, we calculated a value of 5.09 kN for the drag on the nose cone reduction component. The wind load on the handrails was relatively small (0.15 kN), and therefore, these components were excluded from the analysis. The total drag acting on the fish cages (6.9 kN) was also considered by applying an equivalent load to the attachment hinges. Thus, the total drag force acting on the nose cone was 18.6 kN.

Summary of the drag forces. From Eq (9), several drag force calculations were performed considering the characteristics of the water and wind velocities of the potential deployment site. The values were isolated to the specific components of the nose cone and fish cages. Table 2 provides a summary of the loads. Although the initial intent was to use the combination of drag forces to analyze the material stresses under specific loading conditions, this information can also be utilized for the mooring attachment. Since the entire system is to be deployed in a thermal effluent channel with drag due to the water and wind velocities of 2.0 m/s and 14.0 m/s, respectively, the mooring line materials can be specified, given the attachment line configuration.

**Table 2:** Summary of the drag forces acting on the nose cone and fish cage system.

Nose cone		Fish cage	
Wind	Drag force (kN)	Wind	Drag force (kN)
Handrails	N/A	Handrails	N/A
Float	0.38	Float	See footnote “a”
Water		Water^a^	
Plate	5.09	Net (Total)	1.46
Float	6.23	Frame and float	2.54
Total for nose cone	11.7	Total for fish cage	4.0
		Total for 5 fish cages	4.0+4(0.73) = 6.92
Entire system^b^	11.7+6.9 = 18.6		

^a^ The worst-case scenario was assumed with the float components fully submerged.

^b^ Since the floaters were connected butt-to-butt, they were shielded from the flow. This value also assumed the skin friction was minimal and that the drag only impacted the downstream net panels.

**Fig 29:**
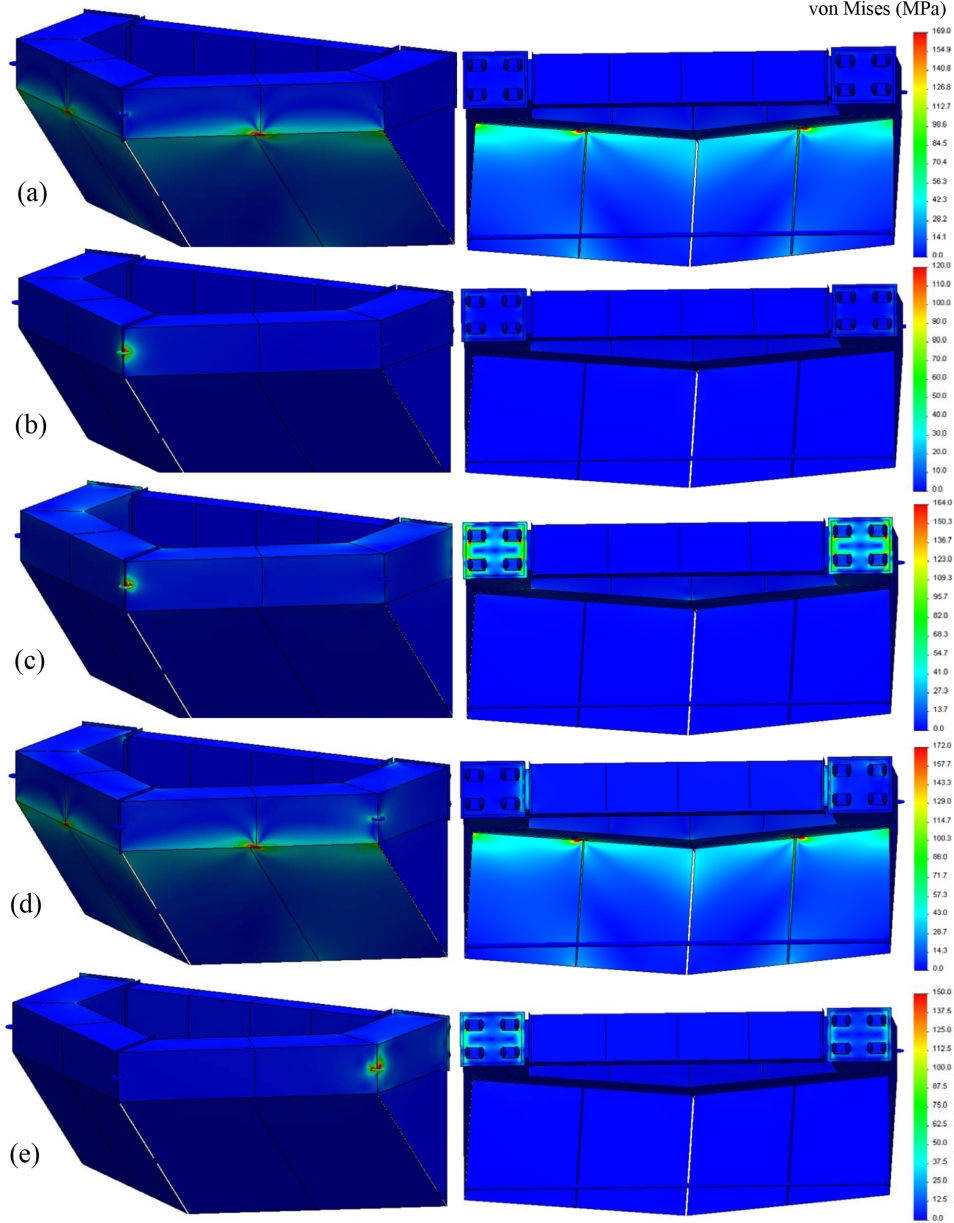
Structural analysis results of the nose cone for load cases (a)–(e). (a) Load case NC-(a): *σ*_99.9%_ = 169 MPa, (b) Load case NC-(b): *σ*_99.9%_ = 120 MPa, (c) Load case NC-(c): *σ*_99.9%_ = 164 MPa, (d) Load case NC-(d): *σ*_99.9%_ = 172 MPa, (e) Load case NC-(e): *σ*_99.9%_ = 150 MPa.

Nose cone results. Fig 29 shows the analysis results, which reveals that the loads associated with case NC–(d) produced the worst-case conditions with the highest magnitude of stresses (*σ*_99.9%_ = 172 MPa). Each sub-figure shows the fore and aft portions of the structure to identify the locations of the highest stress.

**Fig 30:**
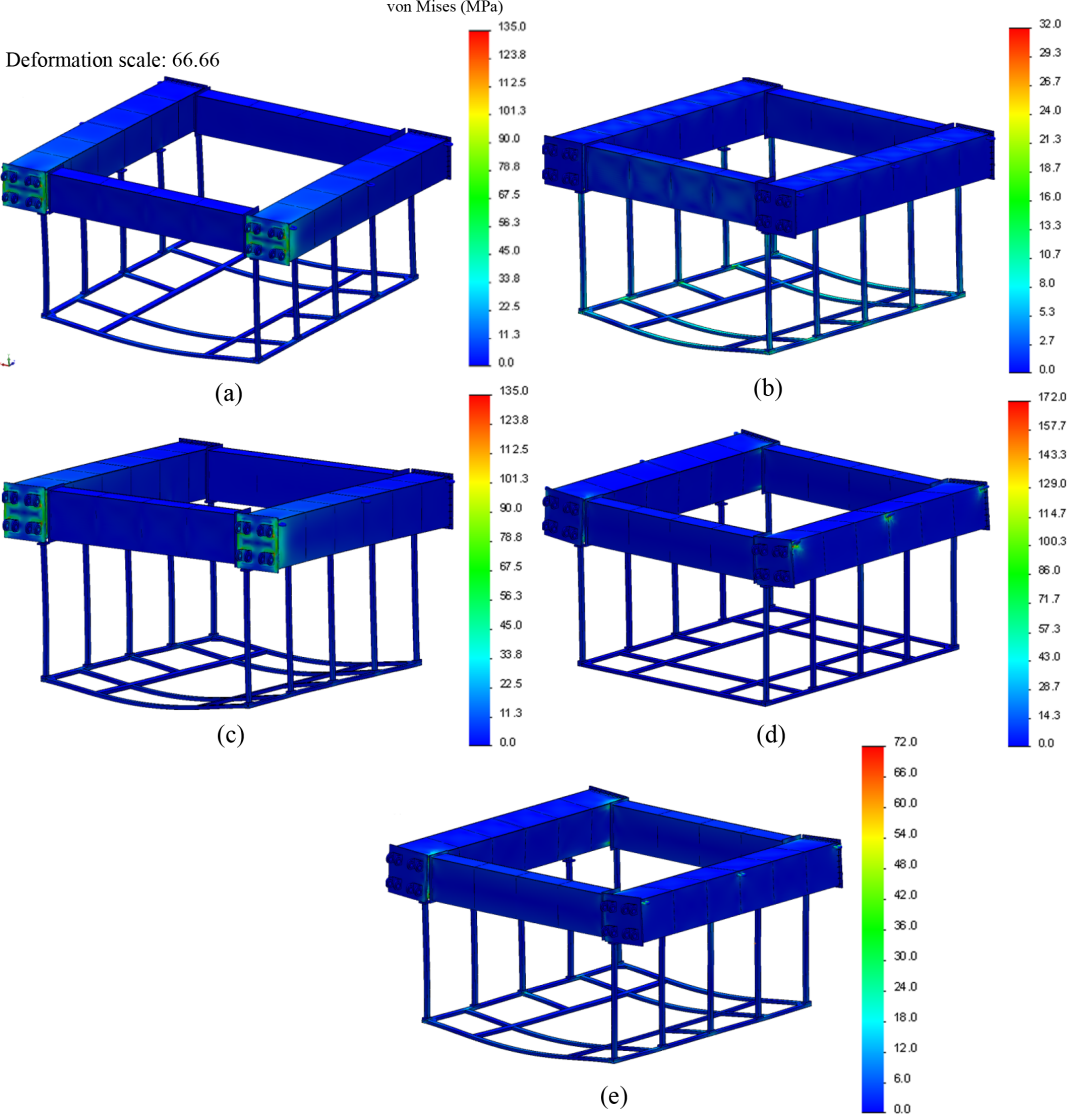
Structural analysis results of cage frame for load cases (a)–(e). (a) Load case FC-(a): *σ*_99.9%_ = 135 MPa, (b) Load case FC-(b): *σ*_99.9%_ = 32 MPa, (c) Load case FC-(c): *σ*_99.9%_ = 135 MPa, (d) Load case FC-(d): *σ*_99.9%_ = 172 MPa, (e) Load case FC-(e): *σ*_99.9%_ = 72 MPa.

Cage frame results. Fig 30 shows the analysis results for each load case of the cage frame, which reveal that load case FC–(d) produced the worst-case conditions with the highest magnitude of stresses (approximately 172 MPa), where the weight of the structure out of the water fully loaded the six lifting brackets. The drag loading associated with load case FC–(c) yielded stresses around the pinned hinges of 135 MPa. If the material considered for these components is a form of mild steel with a yield stress of 248 MPa (an ultimate stress of 8.41 MPa), the safety factor limits can be calculated and assessed.

## Conclusions

The analysis showed that the nose cone generated a reduced current velocity, thus, decreasing the drag forces and the stresses on the effluent channel aquaculture system. The resulting reduced flow velocities of 0.1–0.3 m/s are sufficiently reasonable to choose an appropriate fish species to be raised in the system. The drag on the nose cone and containment modules generated moderate results, such that the resulting stresses were below the yield values. If overhead crane equipment is used, the most critical loading condition will likely occur during the deployment and recovery operations. The design of proper lifting brackets and rigging strategies will be necessary for safety reasons. However, the total drag on the system, estimated at 18.6 kN, can also be used to specify the attachment line components, knowing the geometry and materials. Performing the engineering analyses with a combination of solid modeling, finite element analysis, and CFD tools can be an efficient means of assessing relatively complex component performance from both the structural and fluid interaction perspectives. Subsequent analyses should investigate whether a system like this could be effectively incorporated in the aquaculture industry of a power plant effluent channel.

## Supporting information

S1 Movie Velocity vector field of *x*-*z* plane. (MP4)

S2 Movie Velocity vector field of *x*-*y* plane. (MP4)

S3 Movie Velocity magnitude distribution of *x*-*z* plane. (MP4)

S4 Movie Velocity magnitude distribution of *x*-*y* plane. (MP4)
